# Instantaneous generation of protein hydration properties from static structures

**DOI:** 10.1038/s42004-020-00435-5

**Published:** 2020-12-11

**Authors:** Ahmadreza Ghanbarpour, Amr H. Mahmoud, Markus A. Lill

**Affiliations:** 1 Department of Medicinal Chemistry and Molecular Pharmacology, College of Pharmacy, Purdue University, 575 Stadium Mall Drive, West Lafayette, IN 47906 USA; 2 Department of Pharmaceutical Sciences, University of Basel, Klingelbergstrasse 50, 4056 Basel, Switzerland

**Keywords:** Cheminformatics, Computational chemistry, Structure-based drug design

## Abstract

Complex molecular simulation methods are typically required to calculate the thermodynamic properties of biochemical systems. One example thereof is the thermodynamic profiling of (de)solvation of proteins, which is an essential driving force for protein-ligand and protein-protein binding. The thermodynamic state of water molecules depends on its enthalpic and entropic components; the latter is governed by dynamic properties of the molecule. Here, we developed, to the best of our knowledge, two novel machine learning methods based on deep neural networks that are able to generate the converged thermodynamic state of dynamic water molecules in the heterogeneous protein environment based solely on the information of the static protein structure. The applicability of our machine learning methods to predict the hydration information is demonstrated in two different studies, the qualitative analysis and quantitative prediction of structure-activity relationships, and the prediction of protein-ligand binding modes.

## Abstract (web summary)

Calculating the thermodynamic properties of biochemical systems typically requires resource intensive, multi-step molecular simulations. Here, two deep neural network machine learning methods generate the thermodynamic state of dynamic water molecules in a protein environment solely from information on the static protein structure.

## Introduction

The prediction of thermodynamic properties of biochemical systems such as Gibbs free energies is critical in understanding and quantifying essential biological processes, such as protein folding, protein–ligand and protein–protein binding. Resource intensive molecular simulations are routinely used to sample atomistic configurations of the dynamic biochemical system in order to calculate thermodynamic properties. Recently, machine learning methods have been explored to accelerate and improve configurational sampling of protein systems in comparison to molecular dynamics (MD) simulations^1–8^. This acceleration is achieved by machine learning concepts that learn collective variables from MD trajectories^3,4,7,8^ or that generate new atomistic configurations in a statistically independent manner^1,5,6^. The focus of these methods lies in the thermodynamic characterization for structural studies of proteins. Application of these machine learning approaches to investigate the thermodynamic properties of biochemical processes such as protein–ligand or protein–protein binding is still to be explored.

(De)Solvation of protein and ligand is typically a driving force for such association processes. The thermodynamic properties of water molecules around protein moieties depend strongly on the formation and dynamics of hydrogen-bond networks in a heterogeneous protein environment. Several methods^9^ have been devised to identify water molecules adjacent to proteins’ surfaces which includes knowledge-based methods such as WaterScore^10^ or AcquaAlta^11^, statistical and molecular mechanics approaches such as 3D-RISM^12^ or SZMAP^13^, Monte-Carlo methods such as grand-canonical Monte-Carlo (GCMC) simulations^14^, and MD methods such as WATCLUST^15^, WaterMap^16,17^, or WATsite^18–20^. GCMC- and MD-based hydration-site prediction is accurate and widely accepted as gold-standard to compute the likely water positions in the binding sites of proteins, and the enthalpy and entropy contribution of a replaced water molecule to binding free energies. This statement was confirmed in a recent analysis on the structure-activity relationships for different target systems which demonstrated the superiority of simulation-based water prediction compared to other commercial methods such as SZMAP, WaterFLAP, and 3D-RISM^21^.

Hydration information can be used to estimate the desolvation free energy contributions to a ligand’s binding affinity or the potential for water-mediated interactions^17,22,23^. Grid-based adaptations of the inhomogeneous solvation theory (IST)^24^, for example GIST^25^, have been developed for direct inclusion of the hydration information in docking algorithms.

In addition to water replacement and reorganization, ligand binding typically also involves conformational changes of the protein^26^. Recently, we demonstrated the influence of conformational changes of the protein on hydration-site positions and thermodynamics^27,28^. These studies concluded that hydration-site prediction on flexible proteins needs to be performed on alternative protein states. Furthermore, we recently demonstrated the general importance of water networks around the bound ligand for forming enthalpically favorable complexes^29^. Thus, it is indispensable to re-calculate hydration information in an efficient manner for each bound ligand or even binding pose during docking.

**Fig. 1:**
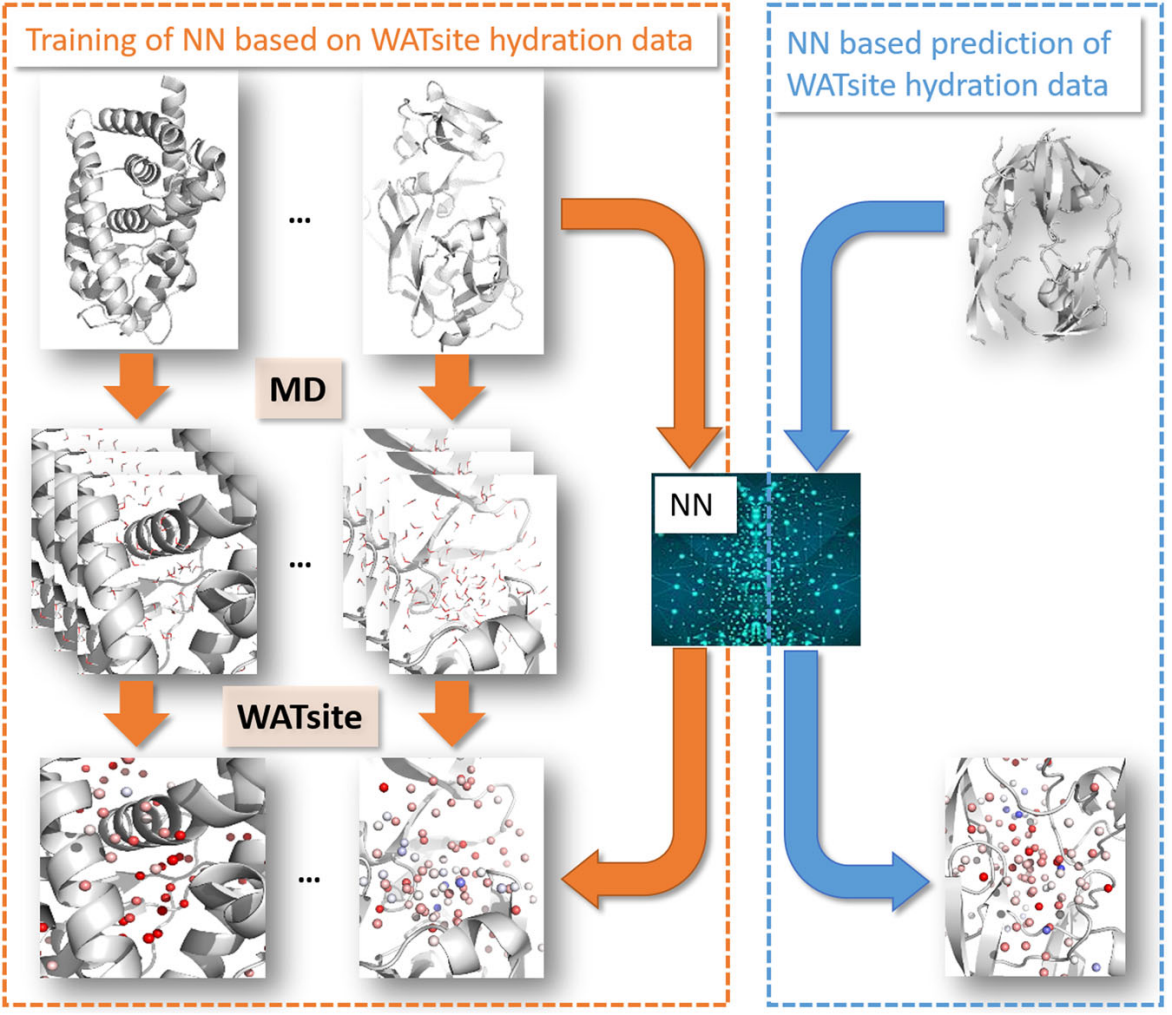
Overall idea of WATsiteOnTheFly. A neural network is trained to generate thermodynamic hydration data based on static protein structure. This allows efficient calculation of (de)solvation data without performing MD simulations.

Hydration-site prediction based on GCMC- and MD simulations is accurate but also rather time-consuming. Utilization of these concepts in a real-world compound-design project on flexible proteins and large sets of ligands with alternative binding poses is therefore difficult to attain with current computer hardware and therefore currently impractical. A significantly more efficient method for hydration profiling is necessary, that would allow its incorporation in virtual screening to dynamic and flexible protein entities. In this study, we provide evidence that modern machine learning approaches may present a realistic solution for obtaining thermodynamic hydration information in an efficient manner; we present the first deep learning methods that instantaneously predict the thermodynamics of hydration data (Fig. 1).

**Fig. 2:**
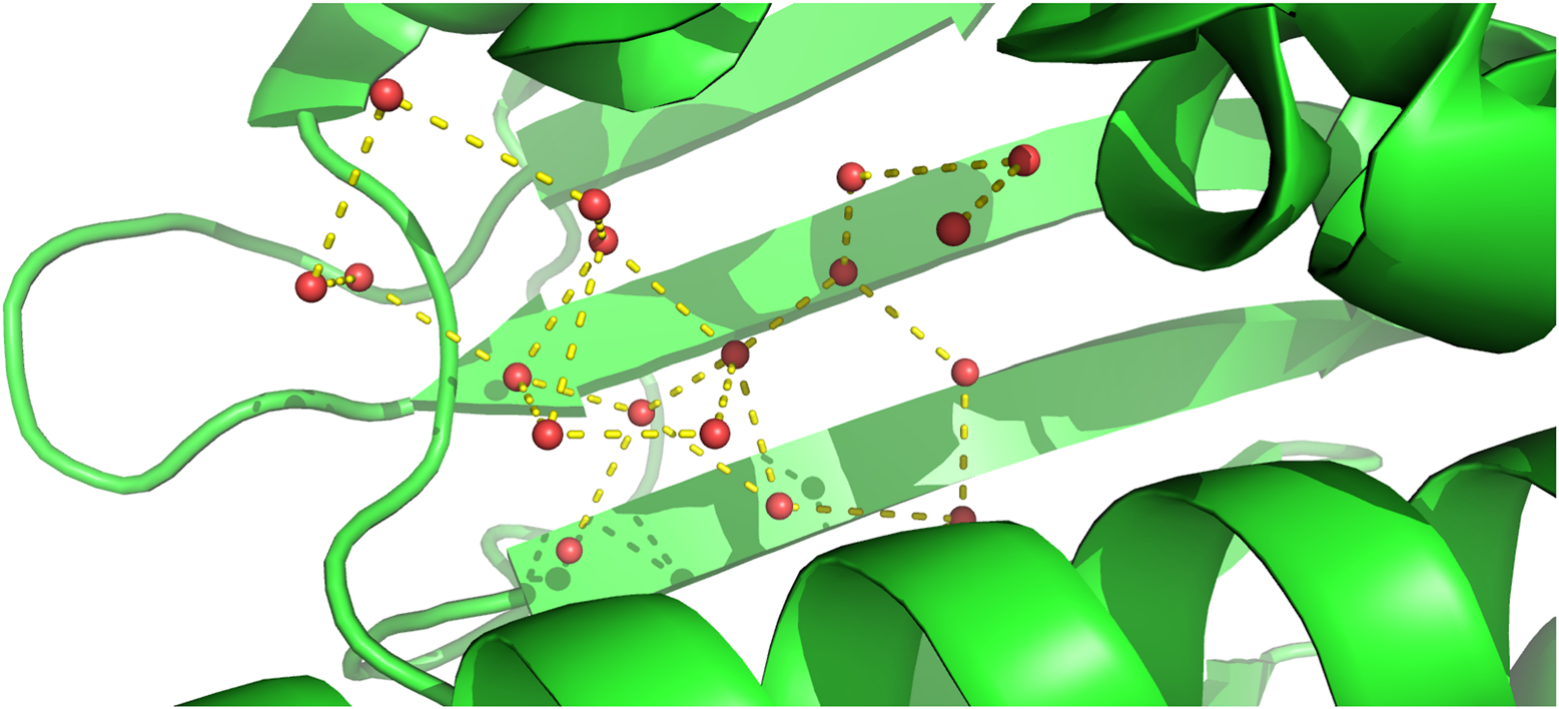
Network of water molecules in binding sites. Example of crystallographic water molecules in the binding site of the apo structure of HSP90 (PDB (Protein Data Bank)-id: 1uyl). As water molecules in the binding site are stabilized by hydrogen-bond interactions to nearby water molecules, models that rely purely on protein–water interactions fail to represent the thermodynamic state and therefore to predict position, enthalpy and entropy of water molecules.

**Fig. 3:**
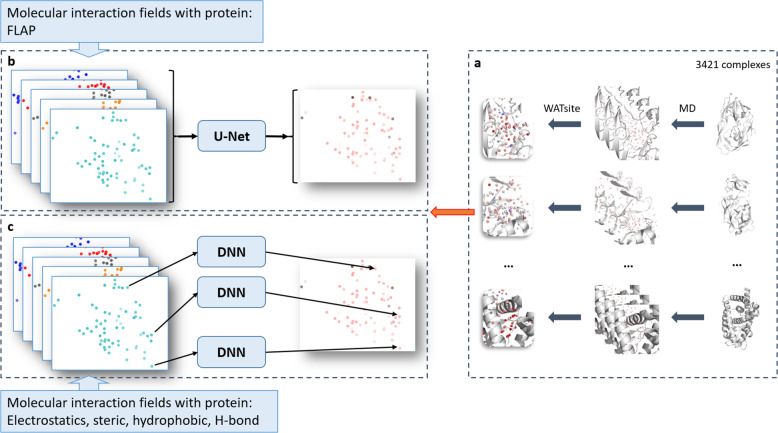
Overall procedure of prediction of WATsite data using neural networks. **a** WATsite simulations are used to generate hydration data. Data is used as output layer for training of neural networks. **b** Direct prediction of complete 3D hydration image using U-Net approach. **c** Point-wise prediction using simple fully connected neural network.

First, we demonstrate that simple machine learning methods based on local descriptors that characterize the direct interaction between protein and a potential water molecule at a specific position in the binding site are insufficient to predict hydration information. The reason for this observation is that interactions among water molecules are critical for stabilizing the hydration pattern in binding sites, forming energetically favorable water networks (Fig. 2). The importance of multi-body effects for the prediction of thermodynamic properties of hydration was also emphasized in previous studies^30^. To correctly model and predict hydration data, more complex machine learning methods need to be designed that include potential water interactions. We have designed two different machine learning concepts based on deep neural networks that include those multi-body effects which are critical to determine the positions and thermodynamic properties of water networks (Fig. 3).

Based on convolutional neutral networks (CNN), the first approach aims to predict hydration information of all grid points in the binding site in a single calculation. First, interactions are computed between protein and multiple atomistic probes placed on a 3D grid encompassing the binding site. Those interaction grids, called molecular interaction fingerprints (MIF), are then used as input to the CNN to predict hydration occupancy. Due to the use of spatial kernels in CNN, correlations between neighboring grid points are incorporated. This allows to implicitly include water–water interactions in the machine learning model.

In contrast, the second model predicts hydration information for each grid point separately using spherical-harmonics local descriptors. Again, interactions between protein and atomistic probes are mapped on a 3D grid. Spherical-harmonics expansions of those interaction maps around each grid point then encode the local environment of a potential water molecule which includes protein–water and water–water interactions.

Both models are trained on a large data set of thousands of protein structures. For each protein structure, MD simulation is performed. Subsequent WATsite analysis predicts hydration density and thermodynamic profiles on a 3D grid. This hydration data on the grid functions as ground truth throughout the training and validation of the neural-network (NN) models. After the model has been trained it can be applied to any static protein structure without the need to prepare and run any MD simulations.

## Results

### Neural network for semantic segmentation

#### Performance in prediction of water-occupancy grids

To incorporate the context of a grid point in the neural network, we utilized CNNs based on the computed MIFs. This approach predicts the water occupancy on a grid point by incorporating spatial context from surrounding grid points during the convolutional feature abstraction process. The CNN network architecture (Supplementary Fig. 3) down-samples the input layer identifying features important for the prediction of water occupancy. The final layers up-sample the grid to the desired occupancy grid. Similar architectures have been used for many applications such as semantic segmentation and generative models. More specifically, we use U-Net as the network architecture. U-Nets are commonly used for semantic segmentation tasks. For image segmentation tasks, a U-Net can rapidly learn to pass critical information such as the outlines of an object, which is similar between input and output layers. This process makes the learning more efficient. Similarly, for the task of water prediction, the surface of the protein is quickly captured by the U-Net from the input data. Our tests showed that without skip-connections, it would be difficult for the network to capture the protein surface, or the solvent accessible surface with the same efficiency.

Initially, we attempted to generate regression models that aimed to predict the actual occupancy value of each pixel or grid point. The resulting models showed poor prediction performance, which can be largely attributed to the highly imbalanced nature of the water grids, i.e., most grid points in a water grid have low or zero occupancy. Alternatively, the water prediction task using 3D CNNs can be tackled as a segmentation problem, detecting dense areas where water is more likely to have high occupancy. We have formulated the problem of predicting water occupancy as a multi-class segmentation problem allowing to identify regions with different levels of water occupancy, here predicting occupancy levels with threshold values of 0, 0.02, 0.03, 0.045, 0.06, and 0.07 (see Supplementary Methods section for details on calculation of occupancy values). The threshold of >0 classifies regions that are generally accessible to water molecules. The threshold of 0.02 represents approximately bulk water density. Occupancy values above this threshold represent regions with increased water density (=hydration sites). Most hydration sites are formed by densities with values between 0.045 and 0.06. Values above 0.07 are rather rare.

To evaluate the neural network’s performance, 5-fold cross-validation was used. The set of proteins was first divided into five groups (Supplementary Data 1). Then, the network was trained on four groups and tested on the one group left out, generating a set of five models. Given the similarity among the proteins in the refined set, we chose not to use random assignment to the five groups. For proper validation of the procedure, we instead minimized the similarity among the different groups by clustering the whole set of proteins based on binding site similarity. This guarantees that during cross-validation, the test set is always the least similar to the training set. To equalize the size of the clusters, samples were removed from larger clusters, resulting in 223 protein systems contained in each cluster. The similarity was calculated using the FuzCav program^31^ and the structures were clustered using the k-modes clustering algorithm^32,33^ on the feature vector generated by FuzCav. For the purpose of data augmentation, the training samples were rotated randomly on-the-fly along the coordinate axes.

**Fig. 4:**
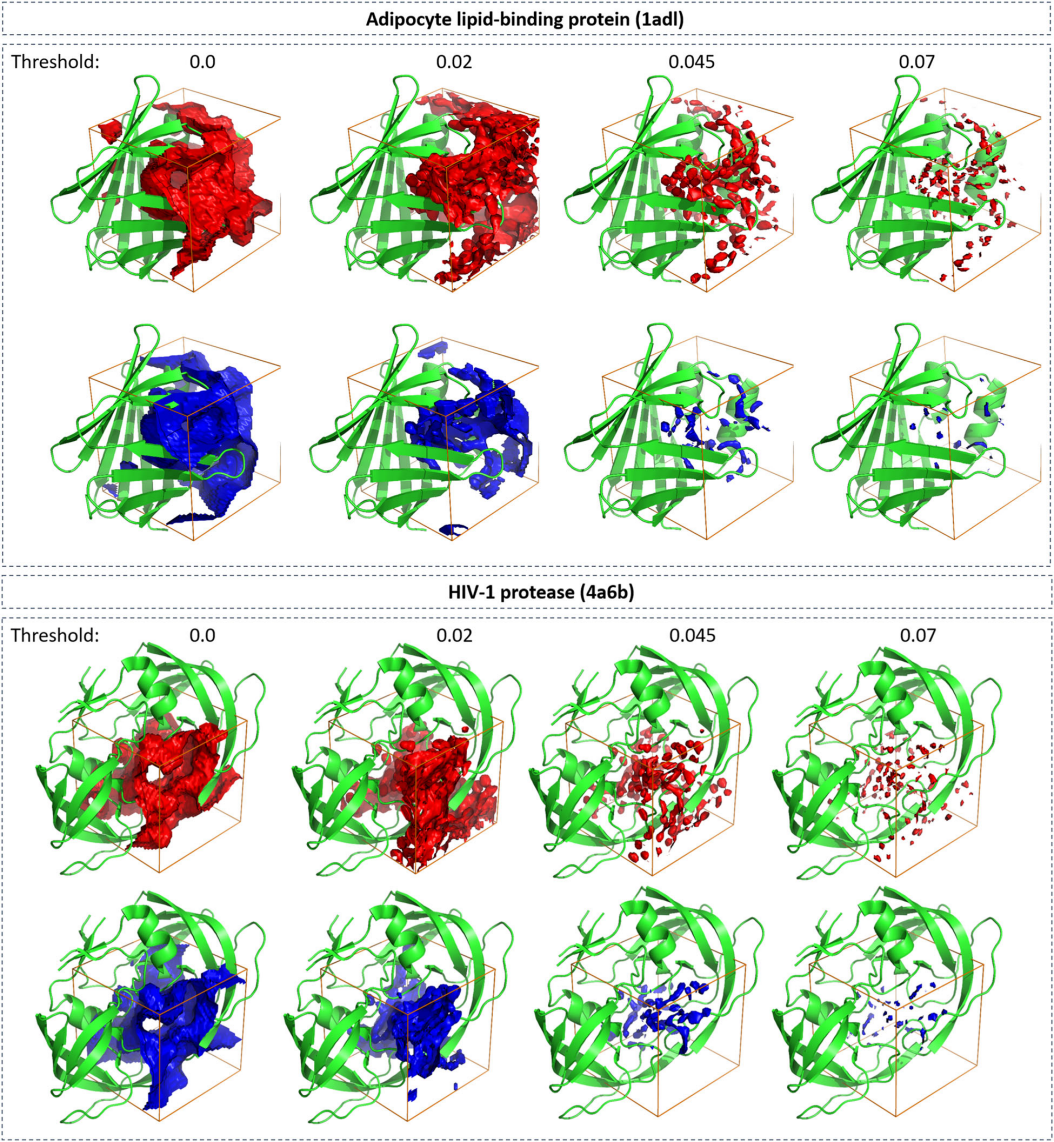
Accuracy of U-Net method. Visual comparison between ground truth (red) and neural-network predicted (blue) water occupancy for adipocyte lipid-binding protein (PDB-code: 1adl) and HIV-1 protease (4a6b). Predictions were performed using U-Net. Isosurfaces at four different threshold values (0.0, 0.02, 0.045, and 0.07) are shown. The task of predicting areas with higher occupancy becomes challenging for the network due to the sparsity of those points (at thresholds 0.045 and 0.07). The regions closer to the corners of the grid are more difficult to predict as information of the context of those grid points is missing.

 Figure 4 shows visualization of the predicted water occupancy for two example proteins at different isovalues representing different thresholds of occupancy. At low thresholds, the quality of predicting occupancies is excellent; predicted and reference occupancy grids largely overlap. As the threshold is increased, the prediction quality drops due to the sparsity of the grid points with high occupancy, demonstrating that even with generalized form of the Dice loss (GDL; see “Methods” for details)^34^ the problem of imbalance in the data set was not completely resolved. We further observed that the network fails to correctly predict the regions close to the boundaries of the grid. A possible explanation for this problem is that for these grid points the network does not receive the full context (MIFs of surrounding grid points) as those neighboring grid points would lie beyond the boundary of the grid box. This failure to correctly predict the occupancy of boundary grid point, however, does not create a serious issue for the purpose of predicting hydration information in the binding site, as the grid points on the boundary of the box lie outside of the binding pocket volume. A mitigation for this problem is to remove the prediction in the boundary regions of the grid box after model generation. Therefore, we focused our analysis on the relevant region in the vicinity of the bound ligand, i.e., all grid points with a maximum distance of 5 Å around the co-crystalized ligand.

 Tables 1 and 2 show different metrics for the prediction quality of the model obtained from the cross-validation. Only data for the left-out systems are used in the statistical analysis. In Table 1, we used smoothed Dice overlap^35^ to measure the overlap between the reference and the predicted grids. In this metric the confidence of prediction of a label is included. For each metric, both the quality of the full grid and for the area within 5 Å from the ligand is displayed. Table 2 displays precision and recall values for the water occupancy in the area within 5 Å from the ligand.

**Table 1:** Performance of different U-Net architectures.

Network	Distance from ligand	Generalized dice loss	Dice overlap (smoothed)
Baseline U-Net	Full grid	0.44 ± 0.08	0.40 ± 0.20
	<5 Å from center	0.35 ± 0.06	0.51 ± 0.17
Inception+Residual U-Net	Full grid	0.29 ± 0.04	0.79 ± 0.04
	<5 Å from center	0.24 ± 0.02	0.84 ± 0.02

Various metrics for the performance of a baseline U-Net and a U-Net using Inception and Residual blocks. Performance on the validation sets are displayed (shown as mean ± standard deviation of cross-validation trials). Metrics are shown for the grids covering the whole binding site and for the sub-grids focusing on the area within 5 Å of the ligand center. The results show that the Inception+Residual U-Net surpasses the baseline model’s performance.

**Table 2:** Precision and recall of convolutional neural network.

Occupancy threshold	Precision	Recall
0.02	0.86 ± 0.03	0.87 ± 0.03
0.03	0.79 ± 0.02	0.81 ± 0.03
0.045	0.73 ± 0.01	0.62 ± 0.03
0.06	0.72 ± 0.01	0.56 ± 0.01
0.07	0.70 ± 0.01	0.54 ± 0.00

Precision and recall values for prediction of WATsite occupancy using fully convolutional neural network (Inception+Residual U-Net) at five different levels of occupancy threshold values.

**Fig. 5:**
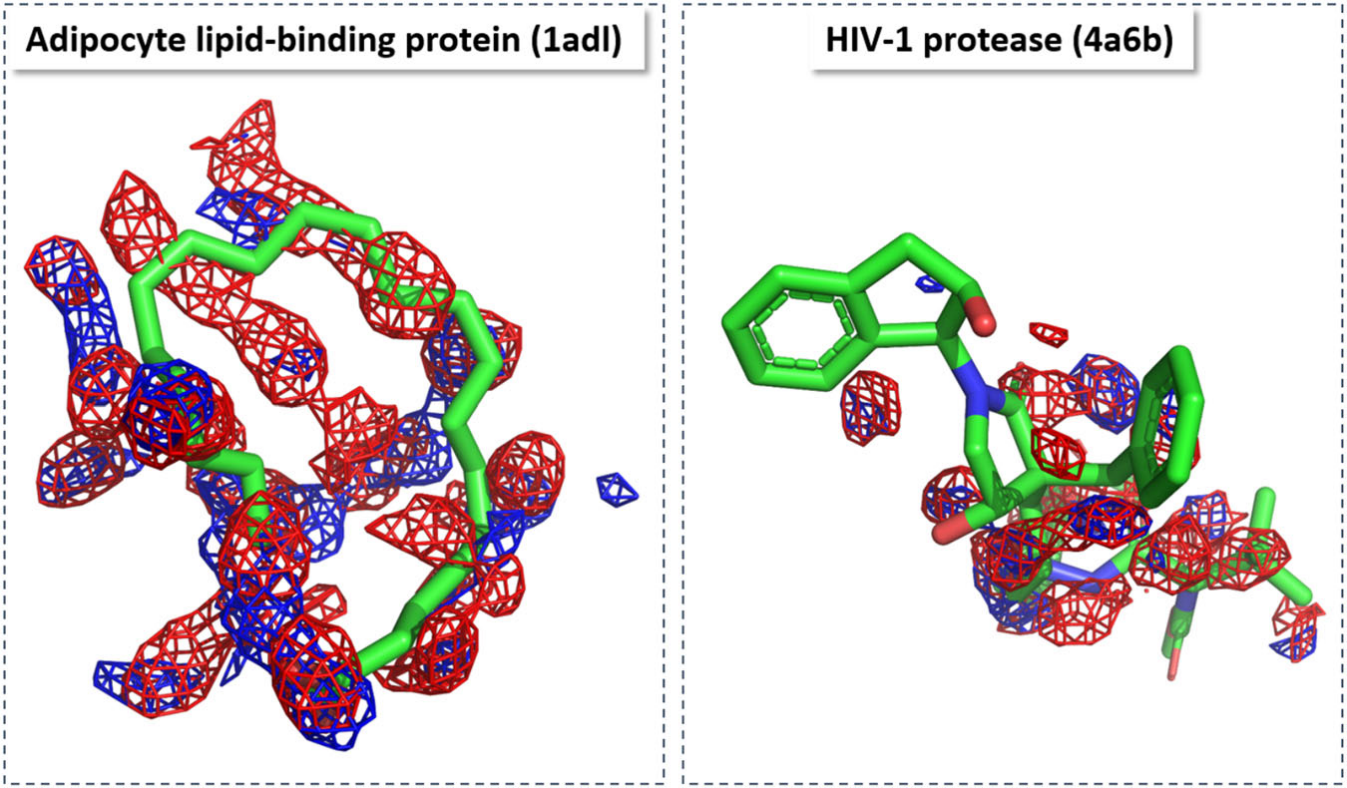
Accuracy of U-Net method focused on binding site. Visual comparison between group truth (red) and neural-network predicted (blue) water occupancy for adipocyte lipid-binding protein (PDB-code: 1adl) and HIV-1 protease (4a6b) within 5 Å of the co-crystallized ligand. Note that the ligands were not included either in the water simulations to produce the ground truth or in the generation of input MIF grids. They were added for visualization purpose only. Predictions were performed using Inception+Residual U-net. Isosurfaces at a threshold value of 0.045 are shown.

 Figure 5 shows an overlay of reference and predicted water occupancies within 5 Å of the co-crystalized ligand to demonstrate the prediction quality in the proximity of the ligand. For applications of the model to drug design, we are interested in this particular region to identify how hydration might enhance, diminish, or interfere with ligand binding at the binding site.

#### Importance of probes

We further analyzed which input MIF grids contributed most to the prediction performance. To compute the feature importance, we used the Mean Decrease Accuracy (MDA) or permutation importance method^36^. This method measures how the absence of a feature decreases the performance of a trained estimator. This method can be directly applied to the validation set without the need of retraining for each feature removal. A feature is replaced with random noise with the same distribution as the original input. One simple way is to shuffle the values of a grid randomly, so that it no longer contains useful information. As expected, the probes which are most influential for the prediction quality were either water probes (OH2) or probes which mediate hydrogen bonding. It should be noted that although water probes from Flap are designed to indicate the water affine areas, they do not linearly correlate with WATsite occupancy, namely, the Pearson correlation coefficient between those MIFs and WATsite occupancy is close to zero. Table 3 shows the performance drop with shuffling of each input grid on the validation sets (sorted by importance of probe).

**Table 3:** Importance of probe grids.

Probe	Dice overlap	Dice overlap (<5 Å from ligand)
**C1=**	0.56 ± 0.06	0.58 ± 0.04
**OH2**	0.51 ± 0.04	0.56 ± 0.03
**CRY**	0.62 ± 0.04	0.67 ± 0.04
**I**	0.63 ± 0.12	0.64 ± 0.09
**O−**	0.71 ± 0.04	0.73 ± 0.02
DRY	0.71 ± 0.07	0.79 ± 0.05
N+	0.77 ± 0.04	0.83 ± 0.02
H	0.75 ± 0.06	0.79 ± 0.02
F3	0.78 ± 0.05	0.83 ± 0.03
OC2	0.79 ± 0.05	0.84 ± 0.02
I-H	0.78 ± 0.05	0.81 ± 0.02
NA+	0.75 ± 0.03	0.81 ± 0.03

Dice overlap value for the cross-validation sets after shuffling of grid point values for each of the 12 MIF grids. The larger the change in value, the more important the probe grid is for the prediction. Important probe grids are displayed in bold. The un-shuffled dice overlap values are shown in Table 1 for all grid points and grid points around ligand.

### Neural networks for point-wise prediction using spherical-harmonics expansion

#### Classification model

In contrast to the segmentation model, in the point-wise model each individual grid point represents a sample that can be used for training and testing of the model. Thus, the size of the data set is significantly increased and allows to design a more aggressive testing protocol compared to the segmentation method. For the point-wise prediction, the same 5-fold splitting procedure of the data set was used. In contrast to the segmentation model, only one-fifth was used for training and four-fifth for testing.

**Fig. 6:**
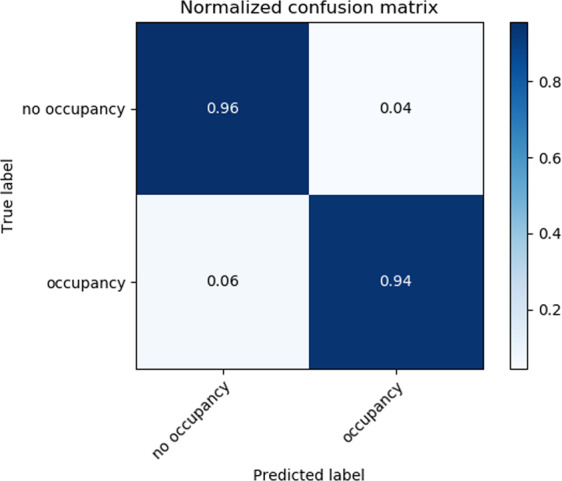
Confusion matrix for classification model. Normalized confusion matrix for classifying grid points with and without water occupancy using neural-network model.

For the classification model, i.e., separating grid points between those with and without water occupancy, the normalized confusion matrix over the test set was computed (Fig. 6). Ninety-four percent of occupied grid points and 96% of unoccupied grid points were correctly classified. The precision values of 0.97/0.92 and recall values of 0.96/0.94 for occupied/unoccupied data signifies the accuracy of the classification model in identifying moieties in the binding site that have been observed to be occupied by water molecules throughout WATsite simulations.

#### Regression model

**Fig. 7:**
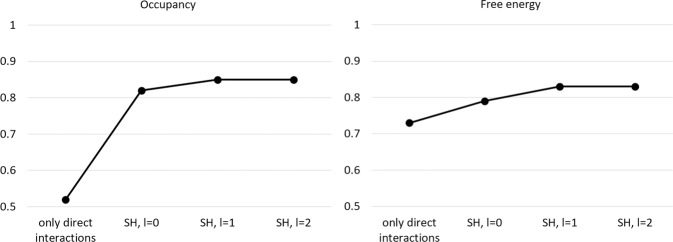
Accuracy of regression model. Regression coefficient *r* for correlating occupancy and free energy values of neural-network predictions with original WATsite data.

Whereas the classification model allows to identify regions with likely water occupancy with high accuracy, a rather small occupancy threshold of 10^−5^ was used. In practice, it is desirable to identify regions in the binding site with high water densities and occupancy peaks that resemble hydration sites. Therefore, a regression model was designed to identify this high density among low-density regions. Using descriptors encoding only the direct interactions with the protein at the specific grid point location (no inclusion of nearby grid points), a mediocre correlation between predicted and ground truth water occupancy was identified (*r* = 0.52) (Fig. 7). Using only the radial distribution of interaction profiles of nearby grid points (l = 0) increases the regression coefficient to *r* = 0.82. Increasing the depth of the spherical harmonics (l = 1) only slightly increases the regression coefficient further to *r* = 0.85. Further addition of angular functions to represent the environmental grid points (l = 2) does not further improve the regression between ground truth and predicted occupancy values. Consequently, we used the regression model with l = 1 for subsequent analysis (see below).

The same trend, although weaker in magnitude, was observed in the regression outcome for the free energy of desolvation at the grid points with occupancy. A maximum *r* value of 0.83 was achieved.

For further evaluation of the neural-network performance, 5-fold cross-validation was used. Again, only a fifth of the data set was used for training in each cross-validation step and four-fifth were used for testing the model. All five models exhibited very similar test set performance. For occupancy the *r* values ranged between 0.85 and 0.86 (standard deviation of 0.004), for free energy it ranged between 0.83 and 0.84 (standard deviation of 0.0044). This highlights the robustness of the model, independent of the specific protein systems used for training.

**Fig. 8:**
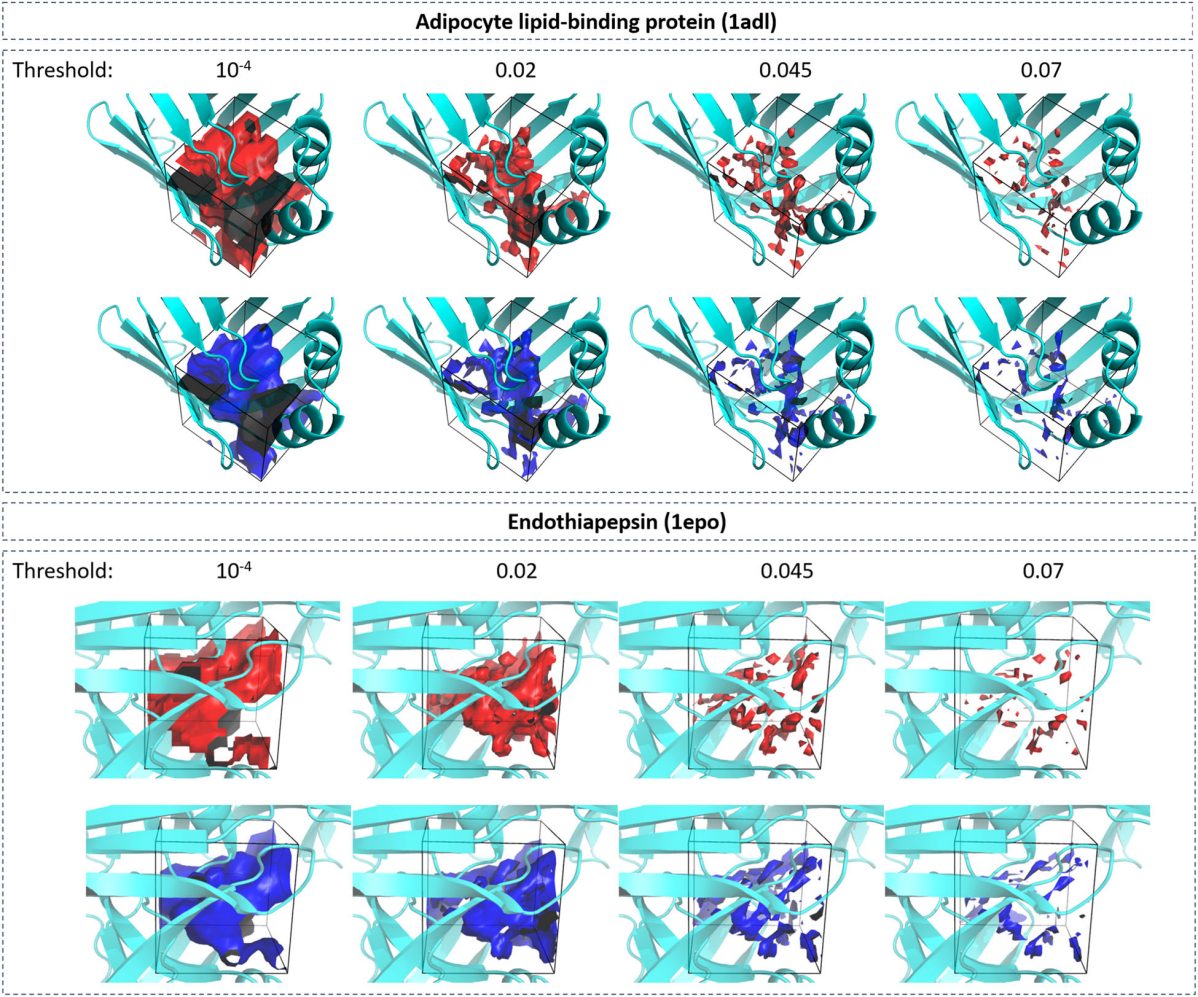
Accuracy of regression model. Visual comparison between group truth (red) and neural-network predicted (blue) water occupancy for adipocyte lipid-binding protein (PDB-code: 1adl) and endothiapepsin (1epo). Predictions were performed using regression neural network. Isosurfaces at four different occupancy values (10^−4^, 0.02, 0.045, and 0.07) are shown.

 Figure 8 shows the comparison of predicted and ground truth water occupancy at isolevels of 10^−4^, 0.02, 0.045, and 0.07 for two different protein systems. Excellent overlap between predicted water occupancy and ground truth was observed with slight deterioration in accuracy for the highest density maps at 0.07. This visual observation can be quantified by measuring the precision and recall values at different classification threshold values of 0.02, 0.03, 0.045, 0.06, and 0.07 (Table 4). Relatively unchanged precision and recall values were observed up to an occupancy threshold of 0.045. Lower accuracy was observed for occupancy values of 0.06 and 0.07. This observation is consistent with previously discussed imbalance between large number of low-occupancy and small number of high-occupancy grid points.

**Table 4:** Precision and recall of regression neural network.

Occupancy threshold	Precision	Recall
0.02	0.79 ± 0.03	0.79 ± 0.06
0.03	0.79 ± 0.04	0.77 ± 0.06
0.045	0.78 ± 0.04	0.76 ± 0.06
0.06	0.75 ± 0.04	0.66 ± 0.06
0.07	0.75 ± 0.04	0.66 ± 0.06

Precision and recall values for prediction of WATsite occupancy using regression neural network at five different levels of occupancy threshold values.

**Fig. 9:**
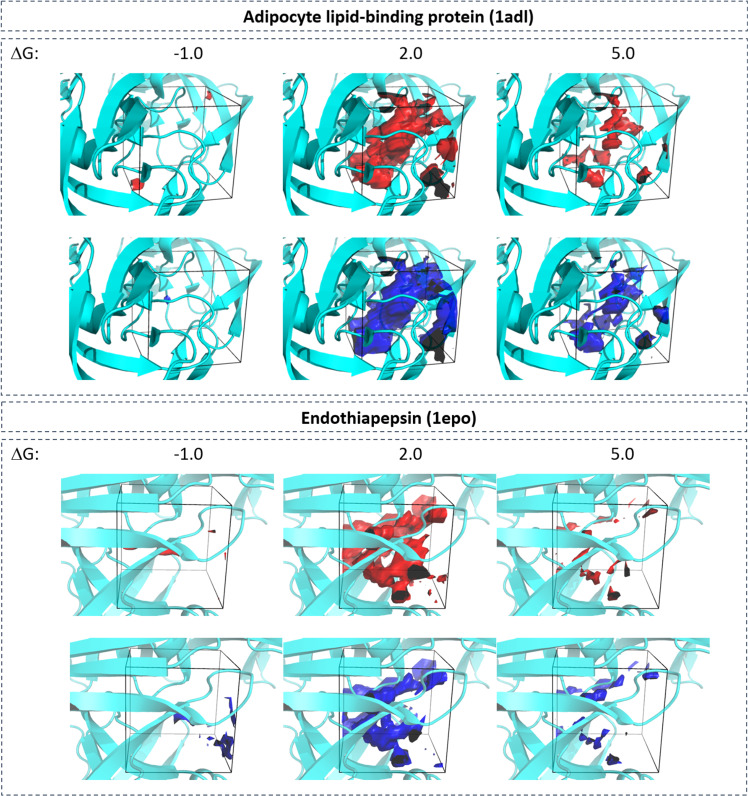
Accuracy of regression model. Visual comparison between group truth (red) and neural-network predicted (blue) desolvation free energy for adipocyte lipid-binding protein (PDB-code: 1adl) and endothiapepsin (1epo). Predictions were performed using regression neural network. Isosurfaces at three different free energy values (−1, 2, and 5 kcal mol^−1^) are shown.

Similar trends were observed for the prediction of free energy values (Fig. 9). Here infrequent negative desolvation values were less accurately predicted compared to positive values. Even regions containing high positive desolvation values were predicted with relatively high quality.

### Comparison with other machine learning approaches

#### Failure of machine learning methods on protein density descriptors

Protein densities distributed on a 3D grid have been used as input descriptors for docking applications^37^. Here, we tested if a similar approach could be used to predict hydration information in the binding site. In detail, an atom is distributed on a 3D grid according to its atom type using a Gaussian distribution function centered on the atom center. Using this Gaussian smearing reduces the sparsity of the input data which would result in poor learning in neural networks since the gradients propagated throughout the network will be sparse as well^38^. Furthermore, Gaussian smearing better represents the spatial extension of the protein and therefore local accessibility of water to the protein surface.

Whereas these input data show good performance for binding pose prediction of chemicals binding to proteins^37^, no significant learning was observed in the context of water-occupancy prediction (data not shown). This failure can be interpreted by the lack of modeling of long-range protein–water interactions and water–water interactions. CNNs based on protein density would allow modeling of local correlation between protein shape/properties and adjacent water occupancy. The stability of water molecules in protein binding sites, however, is strongly influenced by long-range electrostatic interactions and by the formation of hydrogen-bonding water networks^39,40^. Both contributions are difficult to model using localized features extracted by the layers of the CNN.

#### Failure of point-to-point correlations using MIFs

In an another alternative approach, we represented the protein indirectly using molecular interaction fields (MIFs) data^41^. MIFs were generated as described previously. As described in the subsection Probe selection of “Methods”, 12 probes were selected to generate 12 different channels for the input layer. Neural networks were designed for simple point-to-point correlations, where the different MIF input channels were correlated with WATsite occupancy. In our tests, however, neural networks or other machine learning algorithms were unsuccessful in finding any significant point-to-point correlations. From this observation, we concluded that even the MIFs generated with a water probe differ significantly from the WATsite predictions. This can be explained by the fact that the MIFs only represent direct protein–probe interactions and therefore lack the incorporation of water–water interactions. Thus, the interaction value with a probe at a given point does not provide enough information for a network to infer water occupancy. For example, a grid point in an occluded space buried deep inside a protein may have a similar interaction profile with the protein in context of the MIFs to another grid point in a solvent-exposed area. The former point, however, may have lower occupancy due to the lack of stabilizing water–water interactions.

WATsite in contrast includes water–water network interactions explicitly. Furthermore, it explicitly includes entropic contributions, as the water distribution is sampled from a canonical statistical ensemble during the MD simulation. To predict water occupancy at a certain location, the neural network requires not only the interaction information on the corresponding grid point, but also the context of the grid point, i.e., interaction with other water molecules. Those interactions can be represented either by directly including information of neighboring grid points or by the explicit design of input descriptors that include environmental information. The latter approach was described in the section “Neural networks for point-wise prediction using spherical harmonics expansion”, the former was discussed in the section “Neural network for semantic segmentation”.

### Applications

The two NN approaches for the generation of hydration information were applied to three different topics, i.e., the prediction of hydration-site locations in X-ray structures, the qualitative and quantitative analysis of structure-activity relationships (SAR) data, and the improvement of CNN-based pose ranking in docking applications.

#### Prediction of hydration-site locations

**Fig. 10:**
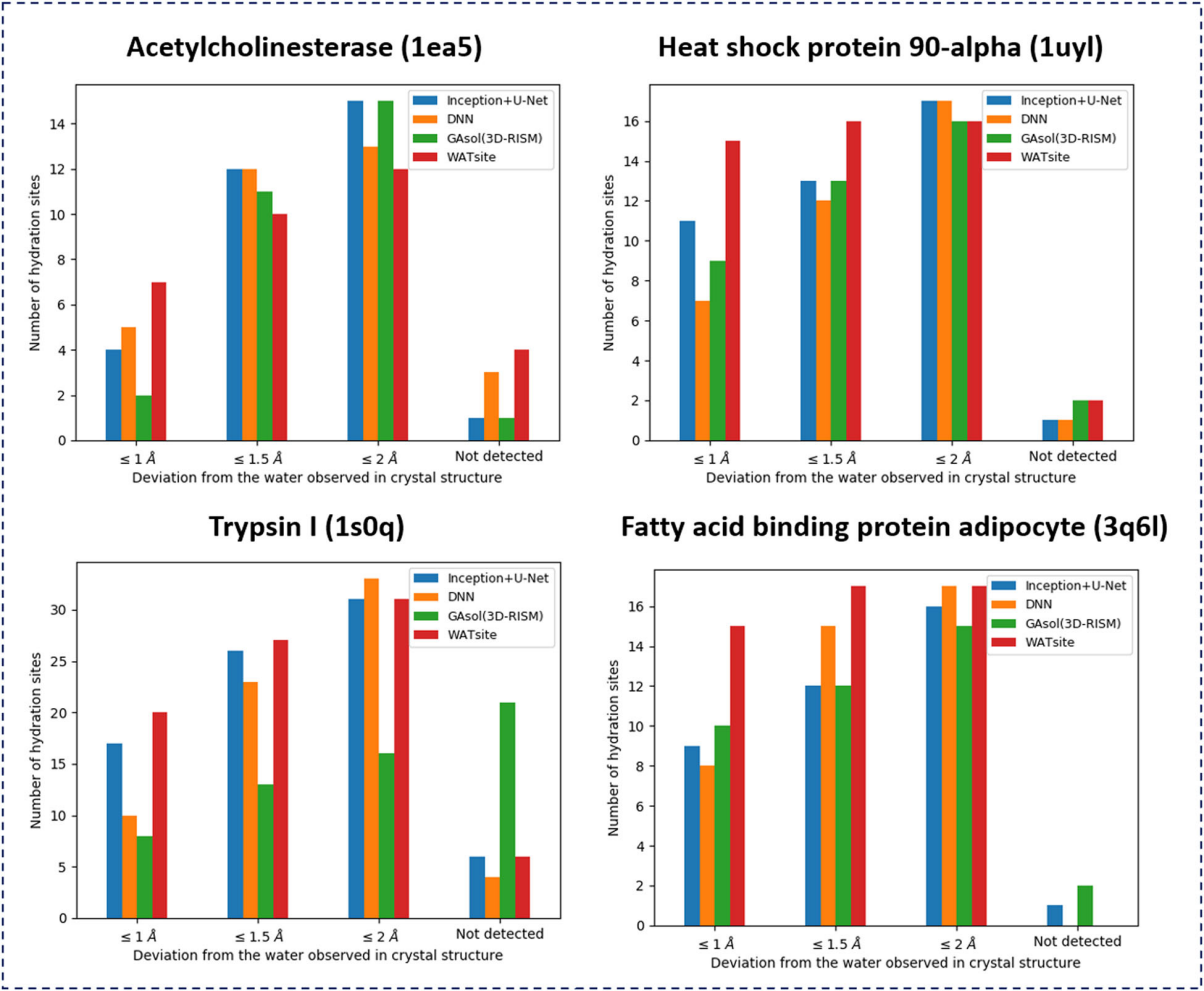
Reproducing hydration sites observed in X-ray crystal structures. Comparison among Inception+U-Net, deep neural network (DNN) based on spherical-harmonics expansion, GAsol/3D-RISM, and WATsite. “Not detected” means no hydration site within 2 Å of X-ray water molecule.

In the first application, we tested the potential of both NN approaches to reproduce the position of crystallographic water molecules in the binding site of four protein systems: Acetylcholinesterase (1ea5), heat shock protein 90-alpha (1uyl), trypsin I (1s0q), and fatty acid-binding protein adipocyte (3q6l) (Fig. 10). Both of our methods were compared to WATsite^18^ and GAsol (3D-RISM)^42^. It should be noted that WATsite had been previously tested to reproduce X-ray water molecules^18,20,27^. We show the prediction performance of finding hydration sites within 1.0, 1.5, and 2.0 Å distance to the corresponding X-ray water location. Hydration sites with distances >2 Å to the corresponding X-ray water locations are considered as failed predictions. WATsite is the most accurate of all methods (Fig. 10), in particular considering small spatial deviations. Both neural networks-based methods either perform equally well or better than GAsol (3D-RISM) and approximate WATsite performance for most systems at a deviation of 1.5 or 2 Å.

It should be noted that a comparison between X-ray water molecules and hydration sites has overall its limitations: First, fit of water positions into electron density obtained from X-ray experiments is not free of errors. Second, X-ray structures are typically resolved at low temperatures underestimating entropic effects. Third, crystal effects may have an influence on water networks, in particular if the binding site is partially or fully solvent exposed. Fourth, the identified hydration sites depend on cluster algorithm and settings, thus adding additional inaccuracies to the grid-based prediction of hydration density. In light of those arguments, we believe the hydration-site predictions using both NN are reasonably accurate, considering their significanty higher efficiency compared to running MD simulations.

#### Structure-activity relationships guided by hydration analysis

Hydration-site prediction using MD-based methods such as WaterMAP or WATsite have been utilized in many recent medicinal chemistry projects to understand ligand binding and structure-activity relationships (SAR), as well as for the guidance of lead optimization. Recently, Bucher et al. demonstrated the superiority of simulation-based water prediction using WaterMAP compared to other commercial methods SZMAP, WaterFLAP, and 3D-RISM^21^ for the analysis of the structure-activity relationships of lead series of different target systems. To demonstrate that the instantaneous prediction of thermodynamic hydration information based on our neural networks can be used with similar confidence in lead optimization projects, we performed three retrospective SAR analyses on heat shock protein 90 (HSP90), beta-secretase 1 (BACE-1), and major urinary protein (MUP).

**Fig. 11:**
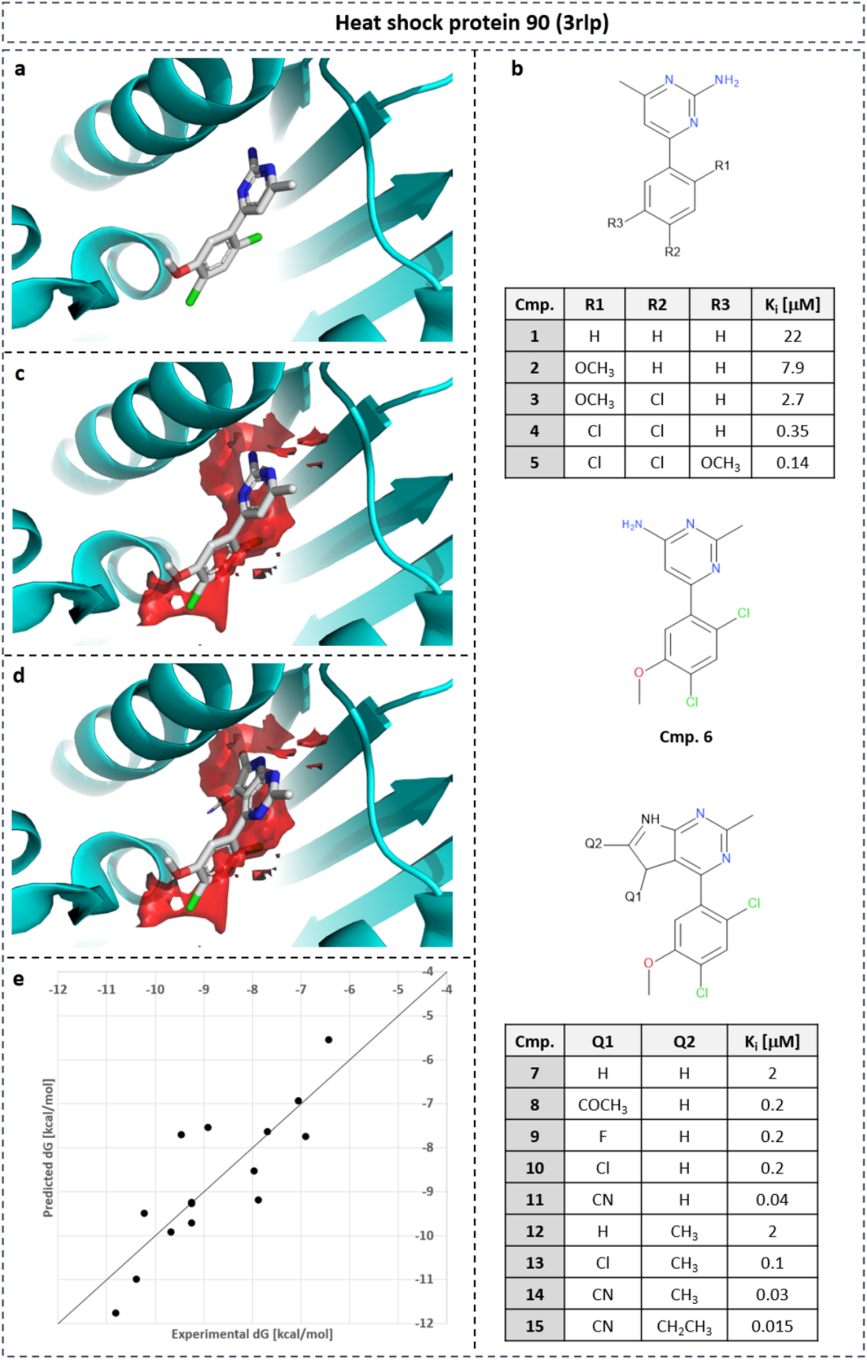
SAR of HSP90 inhibitors. SAR of HSP90 inhibitors guided by gain in desolvation free energy based on point-wise neural-network model. **a** Co-crystalized compound **5** in PDB structure with ID 3rlp. **b** SAR table of 15 inhibitors with substituents replacing water density with unfavorable free energy (c/d: isolevel: 7.5 kcal mol^−1^). **d** Compound 8 from X-ray structure 3rlr. **e** Linear regression between predicted desolvation and experimental binding free energy for SAR series (*r*^2^ = 0.70).

In a study of Kung et al.^43^, a series of HSP90 inhibitors were synthesized and tested (Fig. 11). The design of the molecules was guided by replacing water molecules resolved in the X-ray structure of HSP90. We performed hydration profiling on the X-ray structure 3rlp of HSP90 with the co-crystallized ligand removed using the point-wise neural-network model. Water density with high positive (unfavorable) desolvation free energy (Fig. 11c, red surface, isolevel for Δ*G* = 7.5 kcal mol^−1^) is located around the phenyl ring of compound A (Fig. 11b). Subsequent substitution of hydrophobic groups on the phenyl ring at positions R1, R2, and R3 increases the affinity of the compound from 22 to 0.14 μM by replacing an increasing number of energetically unfavorable water molecules. Additional water density with unfavorable free energy is located adjacent to the pyrimidine ring of the initial scaffold. Extending the pyrimidine scaffold to a pyrrolo-pyrimidine group and adding substituent at Q1 and Q2 position replaces those additional unfavorable water molecules which increases the affinity by almost 10-fold to 15 nM.

Quantitative regression analysis was performed with the aim to correlate desolvation free energy obtained from the point-wise NN with experimental binding affinities. For each ligand atom, the desolvation free energy is computed by trilinear interpolation based on the hydration free energies on the eight grid points that surround the atom. All atomistic desolvation free energies are summed up. Linear regression between desolvation and binding free energy yielded a regression coefficient of *r*^2^ = 0.70 (Fig. 11e).

**Fig. 12:**
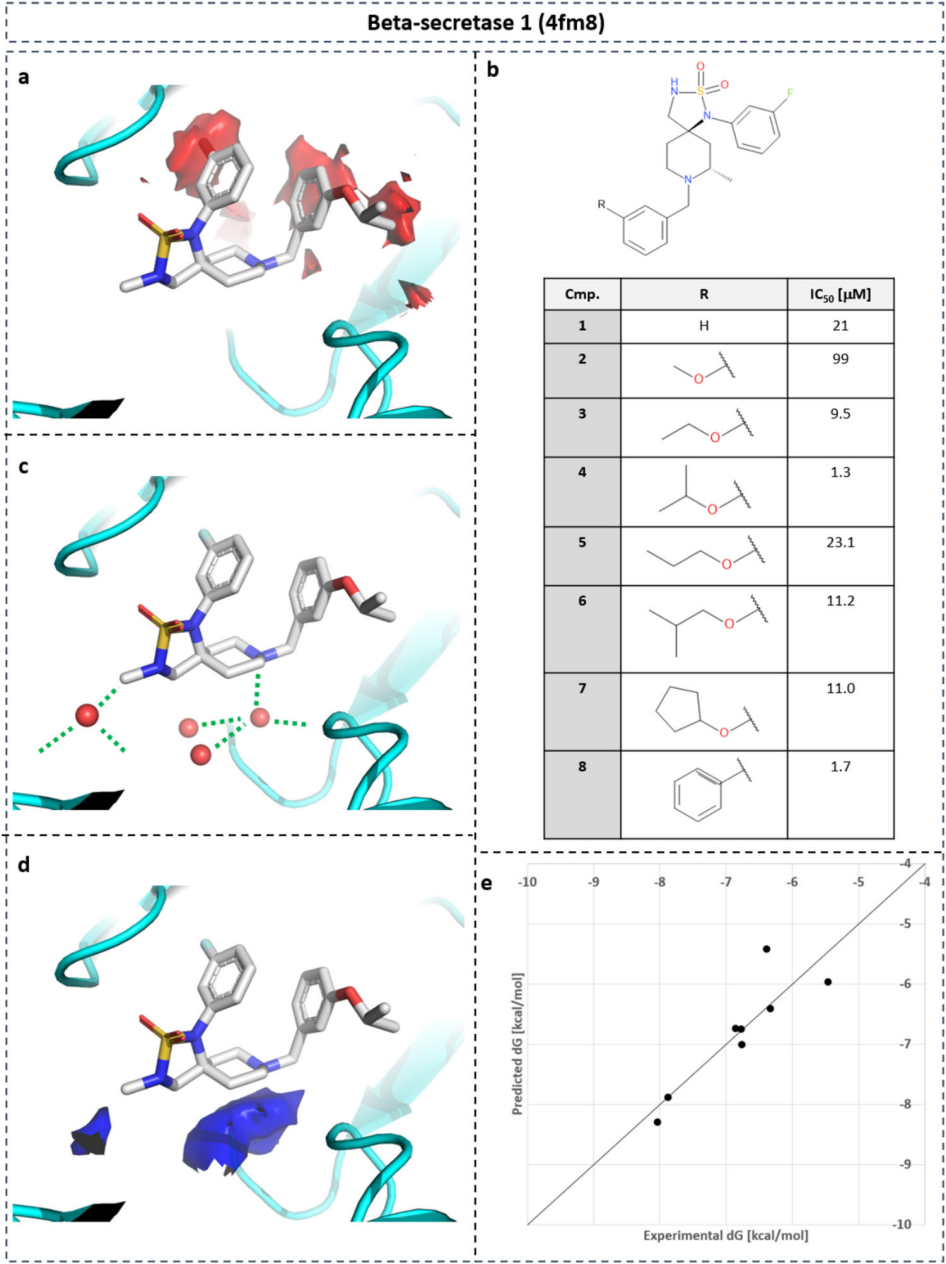
SAR of BACE-1 inhibitors. SAR of BACE-1 inhibitors guided by gain in desolvation free energy based on point-wise neural-network model. **a** Co-crystalized compound **4** in PDB structure with ID 4fm8. **b** SAR table of eight inhibitors with substituents replacing water density with unfavorable free energy (**a**: isolevel: 7.5 kcal mol^−1^). **c** Water-mediated protein–ligand interactions overlap with water density with favorable enthalpy (**d**: isolevel: −3 kcal mol^−1^). **e** Linear regression between predicted desolvation and experimental binding free energy for SAR series (*r*^2^ = 0.78).

A similar retrospective analysis was performed on BACE-1 (Fig. 12). Focusing on the R-group of the terminal phenyl ring (Fig. 12b), density with unfavorable free energy is found adjacent to the R-group (Fig. 12a, red surface on the right). Methoxy substitution (compound **2**) is not able to replace the water density, highlighted by a decrease in affinity. Elongated substituents such as O-ethyl (**3**) and O-isopropyl (**4**) spatially overlap with the unfavorable water density, replacing those water molecules. This results in significant affinity increase from 21 to 1.3 μM. For BACE-1, two regions with favorable water enthalpy were observed (Fig. 12d, blue surface) that coincides with X-ray water molecules (Fig. 12c) which mediate interactions between protein and ligand. Replacement of those water molecules should be considered with great care, as it may lead to a decrease in binding affinity.

Quantitative regression analysis between desolvation and binding free energy was performed for a congeneric series of eight ligands (Fig. 12e). An excellent correlation was obtained with a regression coefficient of *r*^2^ = 0.78. A similar linear regression study on the exact same data set was previously performed using MD-simulation-based hydration-site analysis with WaterMap^44^. This analysis achieved an *r*^2^ value of 0.82. This demonstrates that our NN-based efficient thermodynamic profiling of desolvation is able to generate thermodynamic profiles for hydration comparable to the time-consuming hydration analysis based on MD simulations.

**Fig. 13:**
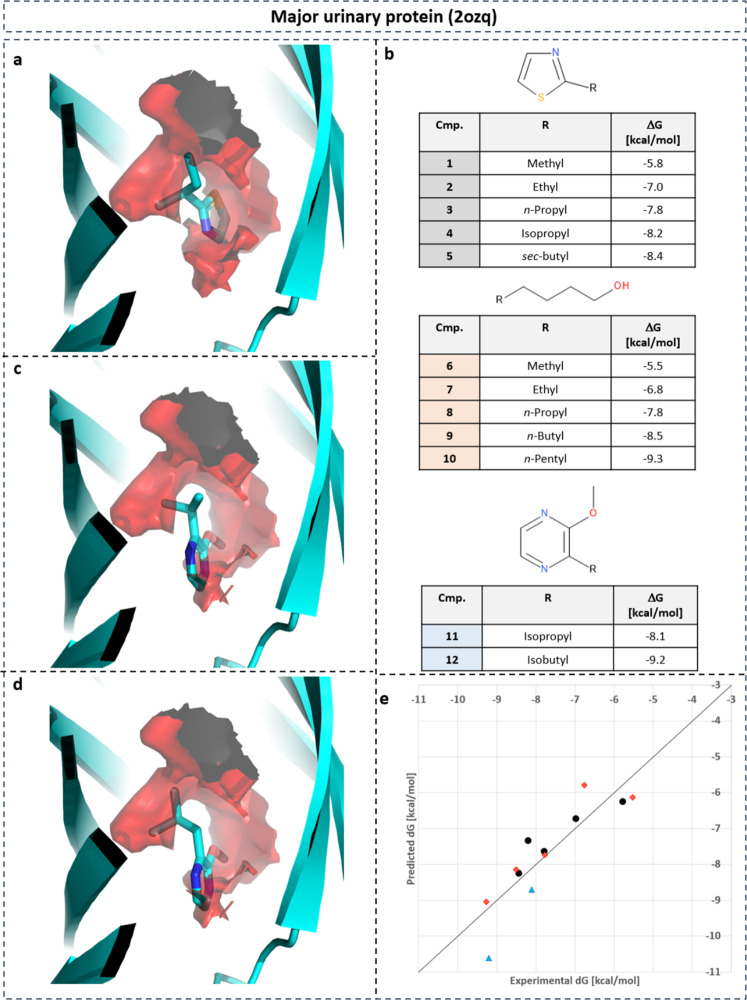
SAR of MUP inhibitors. SAR of MUP inhibitors guided by gain in desolvation free energy based on point-wise neural-network model. **a** Co-crystalized compound **5** in PDB structure with ID 1i06 with water density with unfavorable free energy (isolevel: 8 kcal mol^−1^). **b** SAR table of 12 inhibitors with three different scaffolds and substituents replacing water density with unfavorable free energy. **c** Compound **11** from X-ray structure 1qy2. **d** Compound **12** from X-ray structure 1qy1. **e** Linear regression between predicted desolvation and experimental binding free energy for SAR series (*r*^2^ = 0.77). Compounds **1**–**5** are displayed as black spheres, compounds **6**–**10** as red diamonds, and compounds **11** and **12** as blue triangles.

Retrospective analysis was performed on major urinary protein (MUP) (Fig. 13)^45,46^. The series consists of twelve compounds with three different scaffolds. Figure 13a shows compound **5** in its X-ray structure 1i06. The two terminal methyl groups of the *sec*–butyl substituent overlaps with water density with highly unfavorable hydration free energy. Increasingly smaller substituents display decreasing overlap with positive desolvation free energy grids in agreement with reduced binding affinity. Figures 13c and d display compounds **11** and **12** in their corresponding X-ray structures 1qy2 and 1qy1, respectively. Compound **12** has larger overlap with water density with the most positive desolvation free energy. This results in higher binding free energy compared to compound **11**.

Interestingly, quantitative regression analysis between desolvation and binding free energy revealed that not only an excellent regression within a congeneric series (black spheres: compounds **1**–**5**; red diamonds: compounds **6**–**10**; blue triangles: compounds **11** and **12**) could be obtained but also among all 12 compounds that contain three different scaffolds (Fig. 13e). An excellent correlation was obtained with a regression coefficient of *r*^2^ = 0.77. A similar linear regression study on the exact same data set was previously performed using MD-simulation-based hydration-site analysis with WATsite^20^. This analysis achieved an *r*^2^ value of 0.63. This analysis also demonstrates that our NN-based efficient thermodynamic profiling of desolvation is able to generate thermodynamic profiles for hydration similar to the time-consuming hydration analysis based on MD simulations.

These three examples highlight the potential of our neural-network approach to guide SAR-series expansion by incorporating critical desolvation information including the replacement of unfavorable water molecules and enthalpically favorable molecules which mediate critical protein–ligand interactions.

#### Improved CNN-based pose prediction

In the second application we investigated if the hydration data instantaneously generated by the U-Net neural-network model can be utilized to guide ligand pose prediction. It has been shown previously, that solvent site information generated from MD simulations can assist in detecting protein–ligand interactions and improve docking^47^. Built on these findings, the method AutoDock Bias uses such information to modify and bias the energy terms in order to achieve better performance in docking^48^. Similarly, in our previous study^29^ we showed significant improvement in pose prediction accuracy by adding WATsite occupancy grids as additional input layers to a classification CNN model based on Gnina software^37^. The major issue with this approach is that generating water-occupancy grids for a large data set of protein systems using WATsite or any MD-based water prediction program is computationally expensive. Here, the idea was to investigate if water grids generated via our CNN model can replace the data produced by WATsite to enhance the performance of Gnina.

In Gnina, protein and ligand density are distributed on a 3D grid that encompasses the binding site. For this distribution, a Gaussian distribution function centered on each heavy atom centroid is used. For each atomic element, a separate distribution is computed for protein and ligand. This ensemble of occupancy grids is used as different channels of the input layer of a CNN that classifies native-like poses (RMSD <2 Å) from decoy poses (RMSD >4 Å). Water-occupancy grids predicted by our CNN model were used as an additional input channel to the Gnina CNN.

To provide water-occupancy data for Gnina, we retrained the water predictor network using 2288 and 1133 PDBs for training and test set, respectively. The training and test sets were based on the reduced set from Ragoza et al.^37^. However, we increased the number of bad poses for a more realistic scenario. For each target protein, only one native-like pose with RMSD <2 Å was selected. Since we aimed to utilize the Gnina CNN with and without hydration information for pose reranking, systems with no good poses were removed. The final data set consists of 1394 and 593 protein targets for training and test, respectively. The training was performed for 10,000 iterations. We used the default parameters and the reference model for pose prediction which is made available on Gnina’s Github page (https://github.com/gnina/gnina).

Here, we evaluated the performance of Gnina+water against Gnina alone and Vina/Smina. The results for Vina were obtained from Ragoza et al.^37^.

**Fig. 14:**
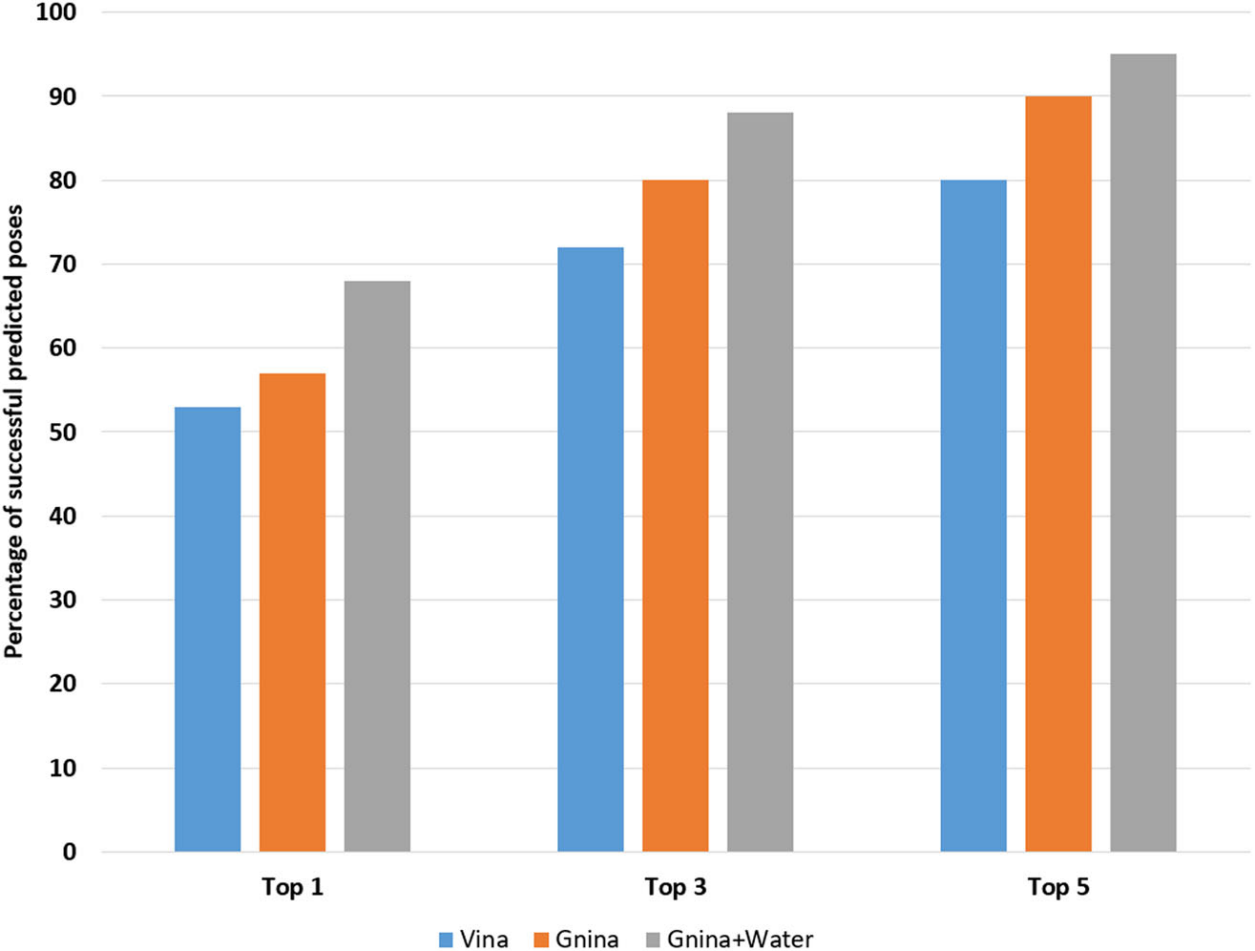
Ranking of docking poses. Percentage of protein systems with native pose (RMSD; <2 Å) in the test set within the top-1, top-3, and top-5 ranked poses using different scoring functions: Vina (blue), CNN with protein and ligand information (orange), and CNN with protein, ligand, and WATsite occupancy information generated by U-Net model (gray).

As it can be seen in Fig. 14, inclusion of hydration occupancy from our neural-network model into Gnina significantly increased the performance of Gnina on the test set.

### Conclusion

Hydration is a key player for biochemical association processes such as protein–ligand and protein–protein binding. The binding partners and the association process itself influence hydration patterns and thermodynamic properties. In order to accurately model hydration in tasks such as flexible protein–ligand or protein–protein docking, the hydration data needs to be computed in an efficient manner without performing time-consuming simulations. In this paper, we demonstrate that instantaneous prediction of thermodynamic properties of biochemical systems is possible due to the development of machine learning algorithms and due to our ability to generate large amount of thermodynamic data. Here, we present the very first deep learning methods to instantaneously predict thermodynamic hydration data, thus providing an efficient alternative to time-consuming MD simulations for the calculation of those properties.

We have developed two alternative deep learning approaches. One method predicts the complete binding site hydration information in a single network calculation in form of U-Net neural networks. The second method relies on descriptors that include potential protein–water and water–water interactions calculated on each grid point. The networks were able to generate precise hydration occupancy and, in case of the point-wise model, also thermodynamics data.

Application of the predicted hydration information to SAR analysis and binding-mode prediction demonstrated the potential of these methods for structure-based ligand design. Future applications include the marriage of protein flexibility and desolvation data in ensemble docking. Due to the efficiency of the methods, precise hydration data could be computed for alternative protein structures, different ligands, and their binding poses in modest computation time, which has been an unfeasible task until now. The routine inclusion of explicit desolvation, water-mediated interactions, and enthalpically stable hydration networks around the protein–ligand complex^29^ may become possible in structure-based ligand design in the near future.

## Methods

### Water prediction on proteins

**Fig. 15:**
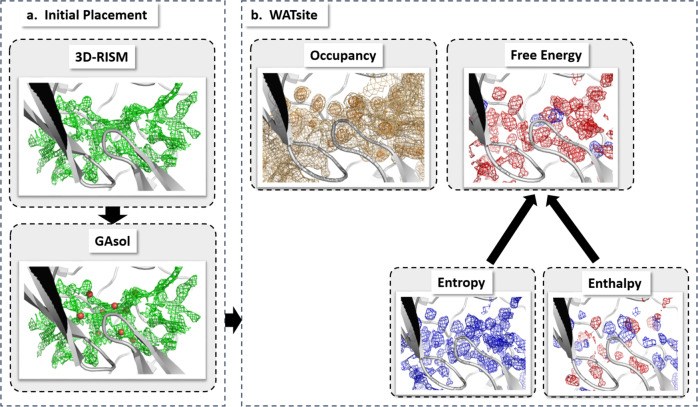
Overall procedure of WATsite. Overall procedure of WATsite combining **a** initial placement of water molecules using 3D-RISM and GAsol, and **b** subsequent MD simulation with explicit water molecules and WATsite analysis to generate water occupancy, enthalpy, and entropy grids (adapted from ref. ^29^).

Here, hydration-site data were generated for several thousand protein systems using WATsite (Fig. 15). The recently published protocol combining 3D-RISM, GAsol, and WATsite (Fig. 15) was used to achieve convergence for hydration-site occupancy and thermodynamics predictions for solvent-exposed and occluded binding sites^20^. Using 3D-RISM site-distribution function^49–51^ and GAsol^42^ for initial placement of water molecules, WATsite then performs explicit water MD simulations of each protein. Finally, explicit water occupancy and free energy profiles of each hydration site (i.e., high water-occupancy spot) in the binding site are computed. This hydration data is distributed on a 3D grid that encompasses the binding site and is used as output layers for the neural networks to be trained on. Details on WATsite simulations and analysis can be found in the Supplementary Methods section.

### Neural networks for WATsite prediction

Two different types of neural networks have been designed to predict hydration information (Fig. 3a). In both approaches, input descriptors were generated for each grid point representing the spatial and physicochemical environment of that potential water location. In the first approach, the complete 3D input grid was translated into a 3D output grid representing the hydration information using a semantic segmentation approach (Fig. 3b). In the second approach, the hydration information of each individual point is predicted based on input descriptors (Fig. 3c).

#### Neural networks for semantic segmentation

In the first approach, to predict hydration data, we adapted deep neural-network concepts commonly used in semantic image segmentation. Semantic image segmentation is the task to identify the pixels in an image that belong to a specific class or category, for example, a specific object in an image. The great advantage of such networks is that they are able to be trained end-to-end by creating a mapping from the input layers to the output images. The resulting output is an image or a grid with the same dimensions as the input layers. Among the various architectures used for this task, U-Net has been demonstrated to often produce superior segmentation performance with smaller training sets compared to other methods^52^. Here, we used different forms of U-Nets but extended the segmentation task to multi-class segmentation. The multiple classes represent the occupancy of water molecules above various threshold values in different moieties along the protein surface.

*Generation of descriptors*. We used the “refined set v.2016” from the PDBind database^53,54^ consisting of 4057 protein–ligand complexes. Hydration-site data was generated using WATsite as described in ref. ^29^ (see also Supplementary Methods). The ligands were removed from their binding site for WATsite calculations but used to define the center of the hydration grids where the center of the grid is aligned to the ligand centroids in the X-ray structure.

All PDB files were processed by removing ions, water molecules, ligands, and other heteroatoms. No proteins with cofactors in the binding site were used in this study. Preparation scripts available in WATsite’s docker image bundle were used to further process the proteins: PROPKA^55,56^ was used for protonation state prediction and LEAP (part of the Ambertools package^57^) for assignment of Amber14 force-field parameters. The prepared protein was used as input for WATsite and for the fully connected network (to generate features with spherical-harmonics expansion method).

For the CNN-based approach, molecular interaction fields (MIF) with different atomistic probes distributed on a 3D grid are used as input. MIFs are generated by first placing a fictitious probe molecule on each point of a 3D grid that encompasses the binding site. The interaction value between probe and protein is calculated at each grid point under the assumption of a rigid protein structure. Instead of providing an image of the protein, this approach rather generates a negative image of it and provides data for the binding site regions of the protein unoccupied by protein atoms but accessible to water molecules.

Molecular interaction fields (MIF) with different atomistic probes distributed on a 3D grid are computed using FLAP^58,59^ and are fed as input descriptors for the CNN. FLAP uses the GRID force field and its own atom types. The internal program GRIN^60,61^ is used to preprocess the protein. Additional details can be found in the Supplementary Methods section. The descriptor grids were aligned and interpolated to the WATsite grids by use of the MDAnalysis package^62,63^. The process for selecting relevant chemical probes for FLAP is further explained in section “Probe selection”. FLAP occasionally failed to generate output for one or two probes for some proteins due to an internal program issue. As this is a commercial software, it was not possible to correct this error. PDB files for which FLAP failed to generate an output were removed. Finally, 3421 PDBs were used for training and testing of the neural-network models (Supplementary Data 2 and 3).

*Probe selection*. In FLAP, MIFs between protein and 78 different chemical probes are generated. To reduce the number of input layers for the CNN model, we performed k-means clustering of the FLAP grids of three randomly selected protein systems. The distance matrix used during clustering was based on Pearson correlation coefficients between the interaction values on the 3D FLAP grids of a pair of probes. In detail, the distance between two interaction probe types was defined as one minus the Pearson correlation coefficient. The number of clusters was chosen to be 12. One representative probe type from each cluster was used to finally generate a set of 12 representative probes with largest diversity between their interaction grids, i.e., smallest Pearson correlation coefficient. These grids represent 12 input channels to the neural network. Increasing the number of channels (probe types) did not lead to significant improvement of the network and only increased the training time.

*Processing of hydration occupancy data*. Initially, the generated neural-network models were designed to generate regression models to predict continuous occupancy values. These models, however, failed due to significant imbalance between low and high-occupancy values (Supplementary Fig. 1). Alternatively, we proceeded with a multi-class segmentation model with six output channels. Each of those channels represents the water occupancy above a chosen threshold. In detail, WATsite occupancy values were transformed into labels based on the threshold values that were selected for the network. The threshold values were 0, 0.02, 0.03, 0.045, 0.06, and 0.07. Input data grids from FLAP were clipped at −20 and 20 kcal mol^−1^ and scaled to be within −1 and 1, to remove the rare, extreme values. This range covers more than 99% of all points (Supplementary Fig. 2).

*Network architecture and model building*. Our neural-network architecture was based on the work in ref. ^64^, with the difference that in our implementation, the network contained six output channels. In detail, a modified version of a U-Net neural network was used which contains Residual connections and Inception blocks. Residual connections were first introduced in ResNets^65^. They have the advantage of preserving the gradient throughout a deep neural network addressing the vanishing gradient problem of those networks.

Another issue is the optimization of the kernel size of the convolutional filters. Sub-optimal kernel sizes can lead to overfitting or underfitting of the network. Inception blocks have been designed to overcome this issue, whereby the Inception blocks contain convolutional layers with different kernel sizes running in parallel. Throughout the training process, the network learns to use the layers with convolutional kernel size that best fits the input data which results in better training process^66^.

**Fig. 16:**
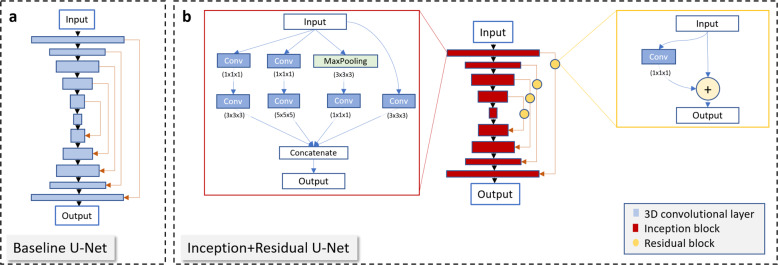
Network architectures. **a** Baseline U-Net and **b** Inception+Residual U-Net architecture used for multi-classification model for hydration density prediction.

The U-Net that we used as a baseline model for our experiments consists of 6 encoder and 5 decoder layers (Fig. 16a and Supplementary Fig. 3a). Each layer has a 3D convolutional layer with kernel size 2, stride size of 2, and zero padding. The number of filters for layers 1–6 is 32, 64, 128, 256, 512, and 512, respectively. Each convolutional layer was followed by a Batch Normalization layer, a Dropout layer, and LeakyReLU activation. Each decoding layer consists of an Upsampling3D layer with size 2 followed by a convolutional layer, Batch Normalization layer, Dropout, and concatenation layer (which provided the skip-connections in the U-Net) and ReLU activation. The number of filters for layers 7–10 is 512, 256, 128, and 64, respectively. The last layer consists of six filters (for the classification of 6 thresholds).

The Inception+Residual U-Net that we used resembles a U-Net, with the exception that each convolutional layer is replaced by an Inception block and the skip-connections contain a Residual block (Fig. 16b and Supplementary Fig. 3b). Inception and Residual blocks and convolutional layers are followed by ReLU activation. The network has 5 encoder layers and 4 decoder layers. All Inception blocks are followed by a Dropout layer. Each decoder layer has an Upsampling3D layer prior to the Inception block. The last layer is a convolutional layer with filter number of 6 and kernel size 1.

As discussed above, regions in the grid with high water occupancy are sparse by nature, resembling a significant imbalance between the number of low-occupancy and high-occupancy grid points. This makes the prediction of higher occupancy grid points difficult, as commonly used loss functions such as mean squared error will not work properly for such imbalanced data. The sparsity of the dense regions causes the network to predict low or zero values for all grid points even for high-occupancy points. This problem also occurs in image segmentation tasks, where the object of interest is small compared to the whole image being analyzed, for example in the detection of small tumors in brain images^34^. One of the loss functions that has been designed to train such imbalanced data is the Dice loss, which is a modified, differentiable form of the Dice coefficient^35^. We used the generalized form of the Dice loss (GDL)^34^ which assigns higher weights to the sparser points:

$${\mathrm{GDL}}=1-2\frac{{\sum }_{l=1}^{6}{w}_{l}\sum _{n}\,{r}_{ln}{p}_{ln}}{{\sum }_{l=1}^{6}{w}_{l}\sum _{n}\,({r}_{ln}+{p}_{ln})}$$

with label weights ${w}_{l}=1/{(\mathop{\sum }\nolimits_{n = 1}^{N}{r}_{ln})}^{2}$ proportional to the inverse of their populations squared. *r*_*ln*_ and *p*_*ln*_ are the reference and predicted label (l) values at a grid point *n*, respectively^67^. This loss function will strongly penalize sparse grid points, enforcing the learning algorithm to more precisely predict those values in addition to the large number of low-occupancy grid points.

Adam optimizer^68^ with learning rate of 0.001 and a batch size of 16 was used for training the model. Learning was performed for 100 epochs using Keras^69^ with Tensorflow^70^ back-end. Once trained, the six output channels of the network are combined to obtain a grid with a range of values which represent the likeliness of hydration.

#### Neural networks for point-wise prediction using spherical-harmonics expansion

In the second approach, the hydration information of each individual point is predicted based on the input descriptors specifying water–protein interactions at this location and the environment of this water location. The approach consists of two subsequent models, a classifier to separate grid point with water occupancy from those without, and a second regression model only for grid points classified as “with occupancy” in the first model. In this regression model occupancy values and free energies of desolvation are computed. In classification and regression model, parameters for the protein atoms such as van der Waals radius and partial charge are directly taken from the coordinate and topology file prepared for WATsite simulations.

*Classification model to identify grid points with water occupancy*. For each grid point, the spatial environment and flexibility of surrounding atoms are computed. In detail, the distance from grid point *k* to all atoms *i* in the neighborhood of the grid point are computed and the van der Waals radius of the protein atom *σ*_*i*_ is subtracted:

1 $${\widetilde{r}}_{ik}=| {R}_{i}-{r}_{k}| -{\sigma }_{i}.$$

All ${\widetilde{r}}_{ik}$ values up to 6 Å are distributed onto a continuous 25-dimensional vector using the Gaussian distribution function, where the value at bin *i* is

2 $${p}_{k,i}=\exp \left(-{\left({\widetilde{r}}_{ik}-\left(i\cdot w-1\mathring{\rm{A}} \right)\right)}^{2}/(2\cdot {w}^{2})\right)$$

with *w* = 7 Å/25. All values are finally scaled using $\tanh ({p}_{k,i}/5)$ to limit values to the range [0;1].

Separate vectors are computed in the same manner for hydrogen-bond donor and acceptor atoms. The motivation for these additional descriptors is that shorter distances between water and hydrogen-bonding groups are observed compared to hydrophobic contacts.

Despite the applied harmonic restraints, dynamic fluctuations of the protein atoms are observed throughout the WATsite MD simulations. These fluctuations can have impact on the accessibility of water molecules to different locations in the binding site. To incorporate those atomic fluctuations in the neural-network predictions of occupancy, we designed a simple flexibility descriptor for the side-chain atoms (backbone atoms are considered rigid in this analysis). The shortest topological distance *t*_*i*_ of a side-chain atom *i* to the corresponding C_*α*_ atom is translated using ${f}_{i}=2\cdot \tanh ({t}_{i}/4)$. The distance between this atom and grid point *k* is then distributed to an additional 25-dimensional vector using a modified Gaussian distribution

3 $${q}_{k,i}={f}_{i}\cdot \exp \left(-{\left({\widetilde{r}}_{ik}-\left(i\cdot w-1\mathring{\rm{A}} \right)\right)}^{2}/(2\cdot {w}^{2})\right)$$

Subtracting this vector *q*_*k*,*i*_ from the unmodified vector *p*_*k*,*i*_ generates a vector that measures the flexibility of the environmental atoms around grid point *k*.

All four vectors are concatenated which generates a 100-dimensional input vector to the neural network for classification.

In addition to the input layer, the neural-network architecture consists of a fully connected hidden layer with 1024 nodes with leaky-ReLU activation and dropout layer with dropout probability of 0.5, followed by a second fully connected hidden layer with 512 nodes with leaky-ReLU activation and a final output layer with sigmoid activation to classify each grid point as either occupied (1) or unoccupied (0). A threshold occupancy value of 10^−5^ in the input was used to separate occupied from unoccupied grid points.

Adam optimizer^68^ with learning rate of 0.001 and a batch size of 250 was used to train the model. Learning was performed for 50 epochs using Tensorflow^70^.

*Regression model*. For each grid point, first the direct interactions between water probe and protein atoms are computed. In detail, electrostatic fields of the protein atoms *i* at location *R*_*i*_ with partial charge *Q*_*i*_ are computed on each grid point *r*_*k*_

4 $${E}_{k}^{{\mathop{{\mathrm{elst}}}}}=\sum _{i}\frac{{Q}_{i}}{| {R}_{i}-{r}_{k}| }.$$

Steric contacts of water probe with protein atoms *i* at location *R*_*i*_ with van der Waals radius *σ*_*i*_ and well-depth *ϵ*_*i*_ is computed using a soft alternative of the van der Waals equation

5 $${E}_{k}^{{\mathrm{sterics}}}=\sum _{i}\sqrt{{\epsilon }_{i}{\epsilon }_{p}}\left({\left(\frac{{\sigma }_{ip}}{| {R}_{i}-{r}_{k}| }\right)}^{4}-{\left(\frac{{\sigma }_{ip}}{| {R}_{i}-{r}_{k}| }\right)}^{2}\right).$$

with *σ*_*ip*_ = *σ*_*i*_ + *σ*_*p*_ (probe *σ*_*p*_ = 1.6 Å) and well-depth of probe *ϵ*_*p*_ = 0.012 kcal mol^−1^. Protein parameters from the Amber14 force field are used.

Hydrophobic contacts are computed^71^ using

6 $${E}_{k}^{{\mathrm{hphob}}}=\sum _{i}\left\{\begin{array}{ll}1\hfill&\,\text{if}\,s\le -1\\ 0.25\cdot {s}^{3}-0.75\cdot s+0.5&\,\text{if}\,-1<s<1\\ 0\hfill&\text{if}\,1\le s.\end{array}\right.$$

with

7 $$s=2.0\cdot \left(| {R}_{i}-{r}_{k}| -{\sigma }_{ip}-2.0\right)/3.0.$$

Hydrogen-bond interactions between water probe and protein acceptor/donor heavy atoms *i* are computed using

8 $${E}_{k}^{{\mathrm{HBond-Acc}}}=\sum _{i}\exp \left(-| {R}_{i}-{r}_{k}-{R}^{0}{| }^{2}\right)$$

and

9 $${E}_{k}^{{\mathrm{HBond-Don}}}=\sum _{i}\left\{\begin{array}{ll}-\exp \left(-| {R}_{i}-{r}_{k}-{R}^{0}{| }^{2}\right)\cdot \cos \left({\alpha }_{iHk}\right)&\,\text{if}\,\cos \left({\alpha }_{iHk}\right)<0\\ 0\hfill&\,\text{if}\,\cos \left({\alpha }_{iHk}\right)\ge 0\end{array}\right.,$$

respectively (*R*^0^ = 1.94 Å).

Each interaction term is then scaled and transformed by a hyperbolic tangent function to the range [0; 1]

10 $${\widetilde{E}}_{k}^{{\mathrm{property}}}=\tanh ({E}_{k}^{{\mathrm{property}}})$$

with the exception of the electrostatic interaction term which is scaled to be within [−1; 1] (small negative van der Waals interaction values are clipped off at zero). Each scaled interaction term is finally transformed into a continuous vector of size 20 using Gaussian distribution functions, where the value at each bin *i* is determined by

11 $${p}_{k,i}^{{\mathrm{property}}}=\exp \left(-{\left({\widetilde{E}}_{k}^{{\mathrm{property}}}-\left(i\cdot w\right.+\min \left({\widetilde{E}}^{{\mathrm{property}}}\right)\right)}^{2}/(2\cdot {w}^{2})\right)$$

(bin width of *w* = 2/20 and *w* = 1/20 for electrostatic interactions and all other interactions, respectively). The five 20-dimensional vectors are concatenated to generate a 100-dimensional input vector to the neural network.

**Fig. 17:**
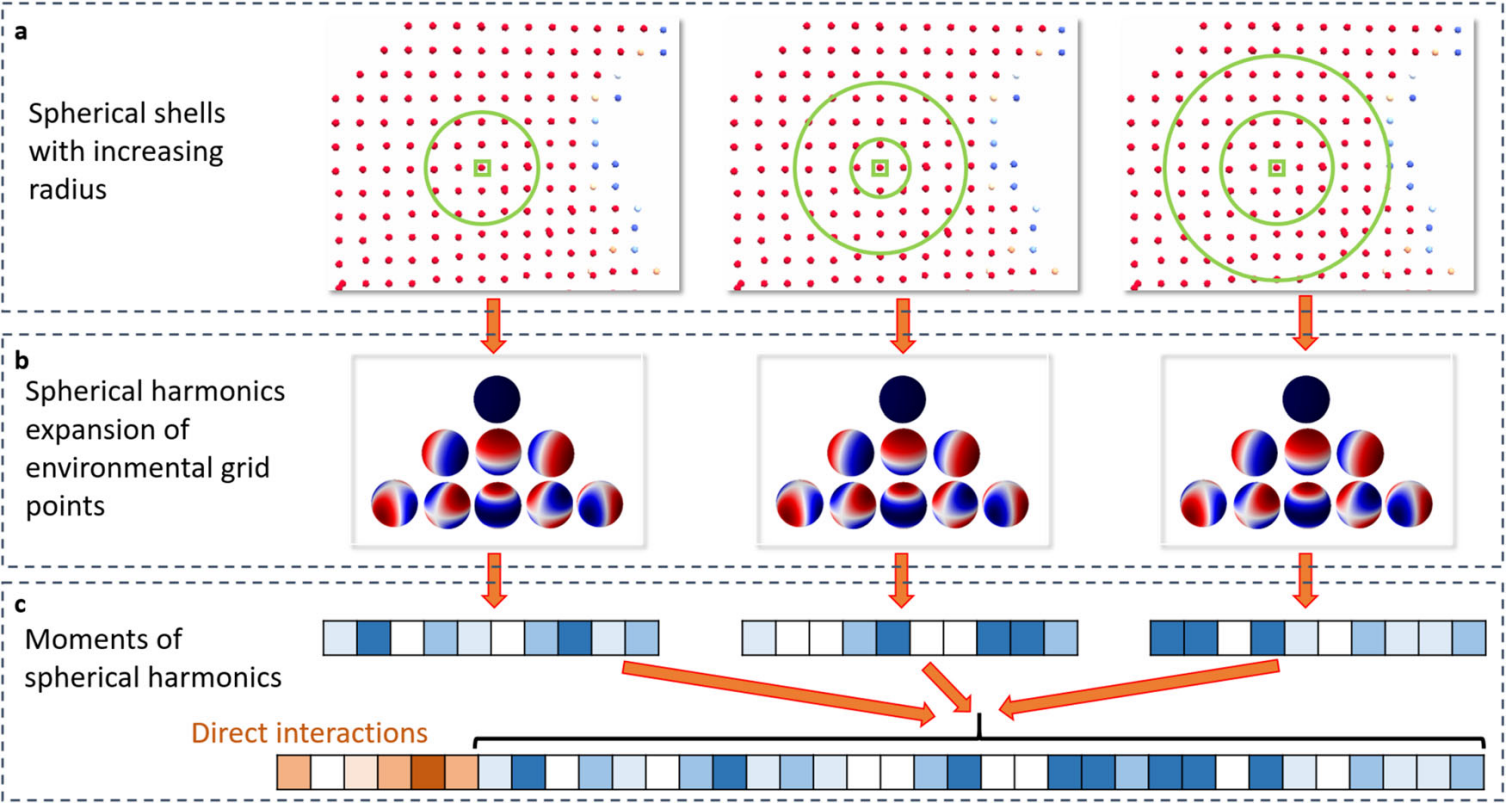
Input of neural network. Generation of input vector for neural network for point-wise prediction of hydration data. **a** For each grid point, the interaction fields from the protein are computed. Nearby grid points within a spherical shell around the grid point are identified. **b** The interaction field distribution of those grid points is represented by spherical-harmonics expansion. **c** The moments of this expansion generate an environment vector. **d** The environment vectors of spherical shells with increasing radius are concatenated together with the direct interaction fields at this grid point. This final vector is used as input for the neural network.

The stability of water molecules not only depends on the protein environment but also on the surrounding network of additional water molecules. Thus, the environment of the water probe needs to be quantified as well. Here, we use a spherical-harmonics expansion of the interaction fields on surrounding grid point as additional descriptors. In detail, seven spherical shells with increasing radius are defined to identify neighboring grid points with increasing distance to probe location: [−*ϵ*; 1 Å + *ϵ*], [0.5 Å − *ϵ*; 1.5 Å + *ϵ*], …, [3 Å − *ϵ*; 4 Å + *ϵ*] (*ϵ* is small value to include grid points with distance at the boundary of interval) (Fig. 17). The grid points in each shell are projected onto a unit sphere and the interaction values of those grid points are used to compute the coefficient of the spherical harmonics up to a certain order *l*_max_:

12 $${\widetilde{E}}_{\,\text{neighbors of}\,k}^{{\mathrm{property}}}(\theta ,\phi )\approx \mathop{\sum }\limits_{l=0}^{{l}_{{\mathrm{max}}}}\mathop{\sum }\limits_{m=-l}^{l}{a}_{l}^{m}{Y}_{l}^{m}(\theta ,\phi )$$

The sum over the degrees of the L2-norm of the coefficients

13 $${\widetilde{a}}_{l}=\mathop{\sum }\limits_{m=-l}^{l}| | {a}_{l}^{m}| |$$

is computed, transformed using $\tanh ({\widetilde{a}}_{l})$ and distributed onto continuous 5-dimensional vectors by a Gaussian distribution function (Eq. (11)). The vectors of direct interactions (Eq. (11)) are finally concatenated with the different coefficient vectors for the different *l* and different interaction types to generate the final input vector to the neural network.

The neural-network architecture consists in addition to the input layer a fully connected hidden layer with 2048 nodes with leaky-ReLU activation and dropout layer with dropout probability of 0.5, followed by a second fully connected hidden layer with 1024 nodes with leaky-ReLU activation and a final output layer with occupancy and free energy values.

Adam optimizer^68^ with learning rate of 0.001 and a batch size of 250 was used for training the model. Learning was performed for 125 epochs using Tensorflow^70^.

### Hydration-site prediction

#### Clustering of occupancy grids to identify hydration sites

To compare hydration occupancy predictions with crystallographic water data and other hydration-site prediction methods, occupancy grids obtained from the two neural-network methods were clustered to predict hydration sites. Two different clustering methods were selected for this purpose. For the Inception+U-Net model, a modified DBSCAN clustering method was utilized (see Supplementary Algorithm 1). For the point-wise prediction model using spherical harmonics, quality threshold (QT) clustering algorithm was used with the following parameters: Maximum cluster diameter: 1.9 Å; minimum number of grid points in a cluster: 5.

#### Evaluation of prediction performance: comparison with experimental data and other hydration-site prediction methods

To evaluate and compare the ability of our methods to reproduce water locations in X-ray data, we chose four apo systems from data from Rudling et al.^72^: Acetylcholinesterase, heat shock protein 90-alpha, trypsin I, and fatty acid-binding protein adipocyte with PDB-ids 1ea5, 1uyl, 1s0q, and 3q6l. All four systems are not part of our training set. The binding site center was defined by superposing the holo form of the same proteins (with ligand present) onto the apo form and using the centroid of the aligned ligand as the center of the grids. We compared the performance of our method with two other methods: WATsite^18^ (MD-based method) and hydration-site prediction generated from GAsol’s clustering method on 3D-RISM grids^42^ (grid-based method). All crystallographic water molecules and ions were removed as part of the protein preparation process. The proteins were prepared automatically by the scripts available in the WATsite 3.0 package for 3D-RISM and WATsite. Both methods were run using their default parameters. The spatial deviation of predicted hydration sites from crystallographic water locations observed in the PDB files was measured. The distance of each crystallographic water molecule to the closest predicted hydration site was measured. Only X-ray water molecules within 5 Å of any ligand and protein atom were considered.

## Supplementary information

Supplementary Information

Description of Additional Supplementary Files

Supplementary Data 1

Supplementary Data 2

Supplementary Data 3

## Data Availability

All data used in this study is based on publicly available protein structure data stored at the PDB database. Cross-validation, training, and test sets are provided as Supplementary Data 1, 2, and 3.

## Code availability

Models, example files and analysis scripts for both neural-network approaches can be found at https://github.com/aghanbar/Watsite_on-the-fly and https://pharma.unibas.ch/de/research/research-groups/computational-pharmacy-2155/research/software.

## References

[1] Noé F, Olsson S, Köhler J, Wu H (2019). Boltzmann generators: sampling equilibrium states of many-body systems with deep learning. Science.

[2] Noé, F., Tkatchenko, A., Müller, K.-R. & Clementi, C. Machine learning for molecular simulation. *Ann. Rev. Phys. Chem.* **71**, 361–390 (2020). 10.1146/annurev-physchem-042018-052331 32092281

[3] Wehmeyer C, Noé F (2018). Time-lagged autoencoders: deep learning of slow collective variables for molecular kinetics. J. Chem. Phys..

[4] Wang Y, Ribeiro JML, Tiwary P (2019). Past-future information bottleneck for sampling molecular reaction coordinate simultaneously with thermodynamics and kinetics. Nat. Commun..

[5] Shamsi Z, J Cheng K, Shukla D (2018). Reinforcement learning based adaptive sampling: REAPing rewards by exploring protein conformational landscapes. J. Phys. Chem. B.

[6] Degiacomi MT (2019). Coupling molecular dynamics and deep learning to mine protein conformational space. Structure.

[7] Chen W, Ferguson AL (2018). Molecular enhanced sampling with autoencoders: on-the-fly collective variable discovery and accelerated free energy landscape exploration. J. Comput. Chem..

[8] Jung, H., Covino, R. & Hummer, G. Artificial intelligence assists discovery of reaction coordinates and mechanisms from molecular dynamics simulations. Preprint at https://arxiv.org/abs/1901.04595 (2019).

[9] Nittinger E (2018). Placement of water molecules in protein structures: from large-scale evaluations to single-case examples. J. Chem. Inf. Model..

[10] Ross GA, Morris GM, Biggin PC (2012). Rapid and accurate prediction and scoring of water molecules in protein binding sites. PLoS ONE.

[11] Rossato G, Ernst B, Vedani A, Smieško M (2011). AcquaAlta: a directional approach to the solvation of ligand-protein complexes. J. Chem. Inf. Model..

[12] Kovalenko A, Hirata F (1998). Three-dimensional density profiles of water in contact with a solute of arbitrary shape: a RISM approach. Chem. Phys. Lett..

[13] Bayden AS, Moustakas DT, Joseph-McCarthy D, Lamb ML (2015). Evaluating free energies of binding and conservation of crystallographic waters using SZMAP. J. Chem. Inf. Model..

[14] Ross GA, Bodnarchuk MS, Essex JW (2015). Water sites, networks, and free energies with grand canonical Monte Carlo. J. Am. Chem. Soc..

[15] López ED (2015). Turjanski. WATCLUST: a tool for improving the design of drugs based on protein-water interactions. Bioinformatics.

[16] Young T, Abel R, Kim B, Berne BJ, Friesner RA (2007). Motifs for molecular recognition exploiting hydrophobic enclosure in protein-ligand binding. PNAS.

[17] Abel R, Young T, Farid R, Berne BJ, Friesner RA (2008). Role of the active-site solvent in the thermodynamics of factor Xa ligand binding. J. Am. Chem. Soc..

[18] Hu B, Lill MA (2014). Watsite: Hydration site prediction program with PyMOL interface. J. Comput. Chem..

[19] Yang, Y., Hu, B. & Lill, M. A. Watsite2.0 with pymol plugin: hydration site prediction and visualization. *Methods Mol. Biol.* **1611**, 123–134 (2017). 10.1007/978-1-4939-7015-5_10 28451976

[20] Masters MR, Mahmoud AH, Yang Y, Lill MA (2018). Efficient and accurate hydration site profiling for enclosed binding sites. J. Chem. Inf. Model..

[21] Bucher D, Stouten P, Triballeau N (2018). Shedding light on important waters for drug design: simulations versus grid-based methods. J. Chem. Inf. Model..

[22] Abel R (2011). Contribution of explicit solvent effects to the binding affinity of small-molecule inhibitors in blood coagulation factor serine proteases. ChemMedChem.

[23] Higgs C, Beuming T, Sherman W (2010). Hydration site thermodynamics explain SARS for triazolylpurines analogues binding to the A2A receptor. ACS Medicinal Chem. Lett..

[24] Lazaridis T (1998). Inhomogeneous fluid approach to solvation thermodynamics. 1. Theory. J. Phys. Chem. B.

[25] Nguyen CN, Kurtzman Young T, Gilson MK (2012). Grid inhomogeneous solvation theory: hydration structure and thermodynamics of the miniature receptor cucurbit [7] uril. J. Chem. Phys..

[26] Lill MA (2011). Efficient incorporation of protein flexibility and dynamics into molecular docking simulations. Biochemistry.

[27] Yang Y, Hu B, Lill MA (2014). Analysis of factors influencing hydration site prediction based on molecular dynamics simulations. J. Chem. Inf. Model..

[28] Yang Y, Lill MA (2016). Dissecting the influence of protein flexibility on the location and thermodynamic profile of explicit water molecules in protein-ligand binding. J. Chem. Theory Comput..

[29] Mahmoud, A. H., Masters, M. R., Yang, Y. & Lill, M. A. Elucidating the multiple roles of hydration for accurate protein-ligand binding prediction via deep learning. *Commun. Chem.* **3**, 19 (2020). 10.1038/s42004-020-0261-x PMC9814895 36703428

[30] Li Z, Lazaridis T (2005). The effect of water displacement on binding thermodynamics: Concanavalin A. J. Phys. Chem. B.

[31] Weill N, Rognan D (2010). Alignment-free ultra-high-throughput comparison of druggable protein-ligand binding sites. J. Chem. Inf. Model..

[32] Huang, Z. Clustering large data sets with mixed numeric and categorical values. in *In The First Pacific-Asia Conference on Knowledge Discovery and Data Mining* 21–34 (1997).

[33] Huang Z (1998). Extensions to the k-means algorithm for clustering large data sets with categorical values. Data Min. Knowl. Discov..

[34] Sudre, C. H., Li, W., Vercauteren, T., Ourselin, S. & Cardoso, M. J. Generalised Dice overlap as a deep learning loss function for highly unbalanced segmentations. *Deep learning in medical image analysis and multimodal learning for clinical decision support* Preprint at https://arxiv.org/abs/1707.03237 240–248 (Springer, 2017). 10.1007/978-3-319-67558-9_28 PMC7610921 34104926

[35] Milletari, F., Navab, N. & Ahmadi, S.-A. V-net: Fully convolutional neural networks for volumetric medical image segmentation. *2016 fourth international conference on 3D vision (3DV)* Preprint at https://arxiv.org/abs/1606.04797 565–571 (IEEE, 2016).

[36] Breiman L (2001). Random forests. Mach. Learn..

[37] Ragoza M, Hochuli J, Idrobo E, Sunseri J, Koes DR (2017). Protein-ligand scoring with convolutional neural networks. J. Chem. Inf. Model..

[38] Kuzminykh D (2018). 3d molecular representations based on the wave transform for convolutional neural networks. Mol. Pharmaceutics.

[39] Breiten B (2013). Water networks contribute to enthalpy/entropy compensation in protein-ligand binding. J. Am. Chem. Soc..

[40] Vaitheeswaran S, Yin H, Rasaiah JC, Hummer G (2004). Water clusters in nonpolar cavities. PNAS.

[41] Artese A (2013). Molecular interaction fields in drug discovery: recent advances and future perspectives. Wiley Interdiscip. Rev.: Computational Mol. Sci..

[42] Fusani, L., Wall, I., Palmer, D. & Cortes, A. Optimal water networks in protein cavities with GAsol and 3D-RISM. *Bioinformatics* **34**, 1947–1948 (2018). 10.1093/bioinformatics/bty024 29346514

[43] Kung P-P (2011). Design strategies to target crystallographic waters applied to the Hsp90 molecular chaperone. Bioorg. Medicinal Chem. Lett..

[44] Brodney MA (2012). Spirocyclic sulfamides as ß-secretase 1 (BACE-1) inhibitors for the treatment of Alzheimer’s disease: Utilization of structure based drug design, watermap, and CNS penetration studies to identify centrally efficacious inhibitors. J. Med. Chem..

[45] Sharrow SD, Novotny MV, Stone MJ (2003). Thermodynamic analysis of binding between mouse major urinary protein-i and the pheromone 2-*sec*-butyl-4,5-dihydrothiazole. Biochemistry.

[46] Malham R (2005). Strong solute-solute dispersive interactions in a protein-ligand complex. J. Am. Chem. Soc..

[47] Arcon JP (2017). Molecular dynamics in mixed solvents reveals protein–ligand interactions, improves docking, and allows accurate binding free energy predictions. J. Chem. Inf. Model..

[48] Arcon JP (2019). AutoDock bias: improving binding mode prediction and virtual screening using known protein–ligand interactions. Bioinformatics.

[49] Kovalenko A, Hirata F (1998). Three-dimensional density profiles of water in contact with a solute of arbitrary shape: a rism approach. Chem. Phys. Lett..

[50] Sindhikara DJ, Yoshida N, Hirata F (2012). Placevent: an algorithm for prediction of explicit solvent atom distribution-application to HIV-1 protease and F-ATP synthase. J. Computational Chem..

[51] Sindhikara DJ, Hirata F (2013). Analysis of biomolecular solvation sites by 3D-RISM theory. J. Phys. Chem. B.

[52] Ronneberger, O., Fischer, P. & Brox, T. U-Net: convolutional networks for biomedical image segmentation. *International Conference on Medical image computing and computer-assisted intervention* Preprint at https://arxiv.org/abs/1505.04597 234–241 (Springer, 2015).

[53] Wang R, Fang X, Lu Y, Wang S (2004). The PDBbind database: collection of binding affinities for protein-ligand complexes with known three-dimensional structures. J. Med. Chem..

[54] Wang R, Fang X, Lu Y, Yang C-Y, Wang S (2005). The PDBbind database: methodologies and updates. J. Med. Chem..

[55] Søndergaard CR, H.M. Olsson M, Rostkowski M, Jensen JH (2011). Improved treatment of ligands and coupling effects in empirical calculation and rationalization of p*K*_a_ values. J. Chem. Theory Comput..

[56] Olsson M, Søndergaard CR, Rostkowski M, Jensen JH (2011). PROPKA3: consistent treatment of internal and surface residues in empirical p*K*_a_ predictions. J. Chem. Theory Comput..

[57] Case, D. A. et al. *Amber 2016 Reference Manual*. University of California, San Francisco, 1–923 (2016).

[58] Baroni M, Cruciani G, Sciabola S, Perruccio F, Mason JS (2007). A common reference framework for analyzing/comparing proteins and ligands. fingerprints for ligands and proteins (FLAP): theory and application. J. Chem. Inf. Model..

[59] Cross S, Baroni M, Goracci L, Cruciani G (2012). GRID-based three-dimensional pharmacophores I: FLAPpharm, a novel approach for pharmacophore elucidation. J. Chem. Inf. Model..

[60] Cruciani, G. *Molecular Interaction Fields: Applications in Drug Discovery and ADME Prediction*, Vol. 1. Vch Verlagsgesellschaft Mbh (2006).

[61] Goodford PJ (1985). A computational procedure for determining energetically favorable binding sites on biologically important macromolecules. J. Med. Chem..

[62] Gowers, R. et al. MDAnalysis: A Python package for the rapid analysis of molecular dynamics simulations. in *Proceedings of the 15th Python in Science Conference* (SciPy, 2016).

[63] Michaud-Agrawal N, Denning EJ, Woolf TB, Beckstein O (2011). Mdanalysis: a toolkit for the analysis of molecular dynamics simulations. J. Comput. Chem..

[64] Tyantov, E. Kaggle ultrasound nerve segmentation competition. https://github.com/EdwardTyantov/ultrasound-nerve-segmentation (2016).

[65] He, K., Zhang, X., Ren, S. & Sun, J. Deep residual learning for image recognition. *Proceedings of the IEEE conference on computer vision and pattern recognition* Preprint at https://arxiv.org/abs/1512.03385 770–778 (2016).

[66] Khan A, Sohail A, Zahoora U, Qureshi AS (2020). A survey of the recent architectures of deep convolutional neural networks. Artificial Intelligence Review.

[67] Crum WR, Camara O, Hill DLG (2006). Generalized overlap measures for evaluation and validation in medical image analysis. IEEE Trans. Med. Imag..

[68] Kingma, D. P. & Ba, J. Adam: A method for stochastic optimization. Preprint at https://arxiv.org/abs/1412.6980 (2014).

[69] François Chollet. Keras. https://github.com/fchollet/keras (2015).

[70] Abadi, M. et al. TensorFlow: large-scale machine learning on heterogeneous systems. tensorflow.org (2015).

[71] Li J (2011). The VSGB 2.0 model: a next generation energy model for high resolution protein structure modeling. Proteins: Struct., Funct., Bioinforma..

[72] Rudling A, Orro A, Carlsson J (2018). Prediction of ordered water molecules in protein binding sites from molecular dynamics simulations: the impact of ligand binding on hydration networks. J. Chem. Inf. Model..

