# Supplementary material for: Instantaneous generation of protein hydration properties from static structures
Source: Commun Chem. 2020 Dec 11;3:188. doi: 10.1038/s42004-020-00435-5 (PMC9814540; doi:10.1038/s42004-020-00435-5)
Supplement: Supplementary file 1 — Supplementary Information [file 42004_2020_435_MOESM1_ESM.pdf]

Supplementary Information

Instantaneous generation of protein hydration properties from static structures

Ahmadreza Ghanbarpour[1], Amr H. Mahmoud[1,2], and Markus A. Lill[*1,2]

[1]Department of Medicinal Chemistry and Molecular Pharmacology, College of Pharmacy, Purdue University, 575 Stadium Mall Drive, West Lafayette, Indiana 47906, United States
[2]Department of Pharmaceutical Sciences, University of Basel, Klingelbergstrasse 50, 4056 Basel, Switzerland.
[*]markus.lill@unibas.ch

November 10, 2020

Supplementary Methods

Molecular interaction fields (MIFs) generation by FLAP

FLAP uses the GRID method to generate Molecular Interaction Fields (MIF) maps using multiple different probes. A probe is the object whose interaction potential energy with the protein target is computed at each grid point. A probe represents a chemical entity, such as an ion, functional group, etc. Each GRID map is calculated by creating a grid around the target molecule (the protein) or specific region (e.g. binding site) and measuring the interaction energy between probe and protein. Hereby, both the probe and the target are immersed in implicit water. The GRID force field uses the concept of "extended atoms", i.e. it combines multiple atoms in a single entity for faster computations. This processing is done using the program GRIN. The pairwise GRID energy for a probe at a given grid point is computed by [1, 2]:

$$E_{PAIR} = E_{LJ} + E_Q + E_{HB} + S$$

where E_{LJ}, E_Q, E_{HB} are Lennard-Jones, electrostatic and hydrogen bond energy terms, respectively. S is an entropic term of the probe at specific grid point.

WATsite simulations

MD simulations were performed with the OpenMM-WATsite package (in house modification of OpenMM) [3]. The protein was described by the AMBER14SB force field and the solvent by the SPC/E water model. Bonds including hydrogen atoms were constrained to their equilibrium lengths using The SHAKE algorithm. Periodic boundary conditions were applied in all three dimensions. Long-range electrostatic interactions were treated with the Particle Mesh Ewald method (cutoff = 10 Å for the direct interactions). Lennard-Jones interactions were truncated at a distance of 10 Å. The system was propagated with a Langevin integrator using a time step of 2 fs and a stochastic thermostat collision frequency of 1 ps^{-1}. Constant pressure was controlled by adjusting the isotropic box size using MC moves every 25 time steps. Each system was first energy minimized. After heating to 298 K over a 50 ps MD simulations, the system was equilibrated by 1 ns of MD simulations at a temperature of 298 K and pressure of 1 bar. All protein heavy atoms were harmonically restrained during the minimization and equilibration process with a spring constant of 4.8 kcal $Å^2$ mol^{-1} . Finally, MD simulation was performed over 20 ns, which allowed enough time for the hydration site prediction to converge [4].

WATsite analysis on grid

Each MD trajectory was analyzed using WATsite3.0 [5] where occupancy, enthalpy and entropy were computed in a 3D grid with spacing of 0.5 Å encompassing the binding site. The same protocol that was originally developed for hydration site analysis [6, 7], was applied for hydration analysis on grids: Occupancy of water molecules is distributed onto the 3D grid with a Gaussian distribution function centered on each water's oxygen atom. In contrast to standard WATsite, no clustering into hydration sites is performed. Each grid

point is considered as a 'pseudo-hydration site'. Throughout the occupancy, enthalpy and entropy calculation, any water molecule within 1 Åradius of a specific grid point throughout the MD trajectory contributed to the calculation of the hydration properties on this grid point. The desolvation enthalpy and entropy of the 'pseudo-hydration site' is calculated similarly as in the original hydration site analysis [6, 7].

Clustering algorithm for generating hydration sites

Supplementary Algorithm 1: Clustering algorithm for identifying hydration site location from output occupancy of Inception+U-Net model

```
Input: Set of grid point coordinates: gridPoints; clustering parameters: threshold = 3.0, eps = 0.5,
       minSamples = 5, start = 0.5, end = 1.8, stepSize = 0.05
Output: Set of cluster center coordinates
Function MergePoints(points,cutoff):
    Construct distance matrix of points
    Use the distance matrix as adjacency matrix to construct undirected weighted graph G
    mergedPoints = [ ]
    foreach edge in G do
        if edge.weight > cutoff then
            Delete edge from G

    Find connected components of graph G and generate each as a subgraph and construct set of
     subgraphs G_s
    foreach subgraph in G_s do
        Average points coordinates to get a single coordinate value (x,y,z)
        Append (x,y,z) to mergedPoints

    return mergedPoints

Function StepwiseMerge(points, start, end, stepSize):
    cutoff = start
    while cutoff ≤ end do
        points = mergePoints(points,cutoff)
        cutoff = cutoff + stepSize

    return points

Function clusterPoints(gridPoints, threshold, eps, minSamples, start, end, stepSize):
    Discard points in gridPoints lower than threshold and retain the remaining in set points
    labels = DBSCAN(points, eps = 0.5, minSamples = 5)
    Find center coordinates of each cluster label by averaging and create a new points array append all
     centers including the points not belonging to any cluster
    clusteredPoints = stepwiseMerge(points, start, end)
    return clusteredPoints
```

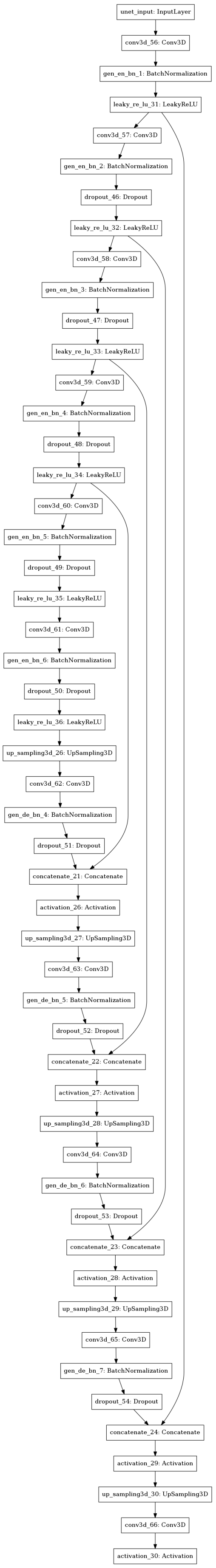

Supplementary Figure 1: Baseline U-Net

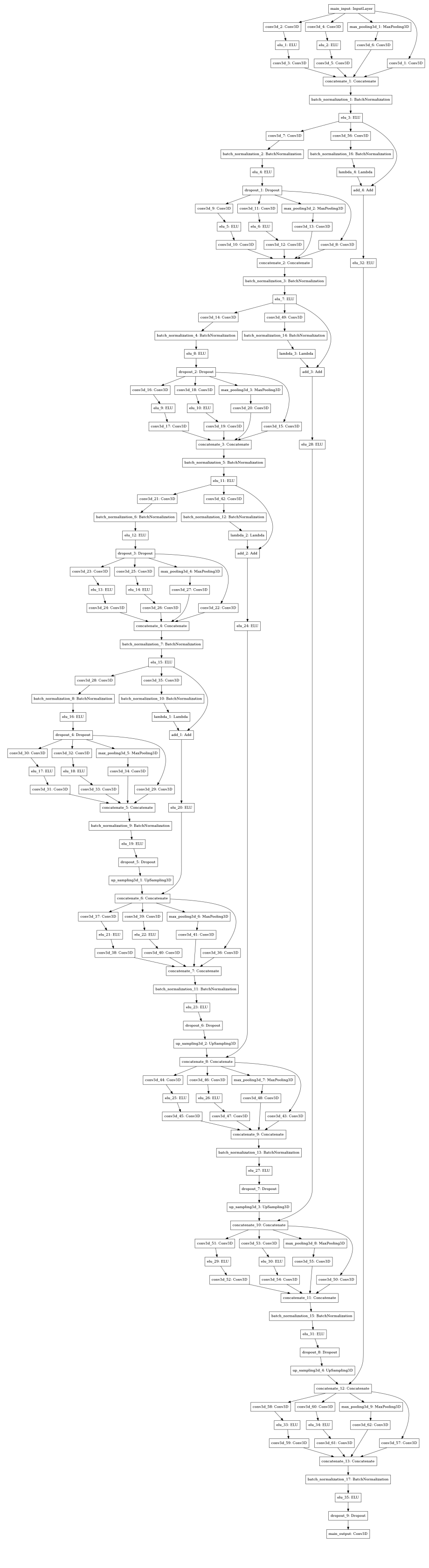

Supplementary Figure 2: Inception+Residual U-Net

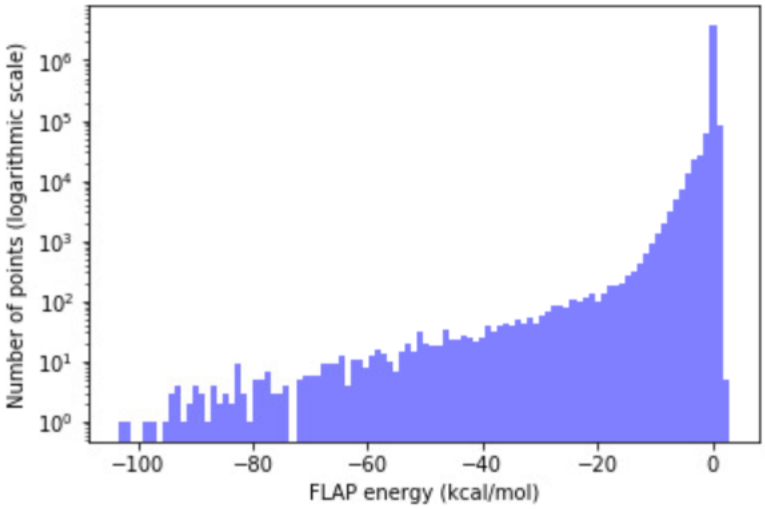

Supplementary Figure 3: Distribution of values for all 12 Flap grids of three example structures (combined): 4bkt, 3b7r and 5c5t. Note the logarithmic scale on the y axis. 99 percent of the points are larger than -20 kcal mol^{-1}.

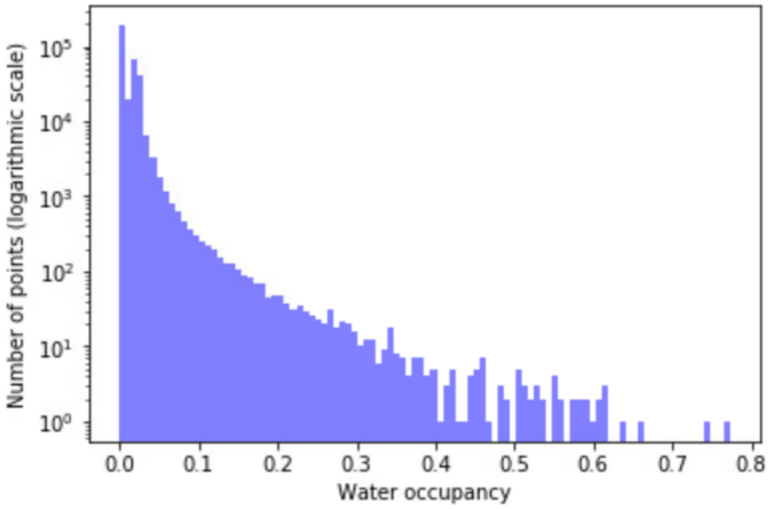

Supplementary Figure 4: Distribution of values for WATsite occupancy grids of three example structures (combined) shown in logarithmic scale: 4bkt, 3b7r and 5c5t. The figure shows the severe imbalance between the high occupancy data points and the lower ones.

Supplementary References

[1] Peter J Goodford. A computational procedure for determining energetically favorable binding sites on biologically important macromolecules. *J. Med. Chem.*, 28(7):849–857, 1985.

[2] Gabriele Cruciani. *Molecular interaction fields: applications in drug discovery and ADME prediction*, volume 1. Vch Verlagsgesellschaft Mbh, 2006.

[3] Ying Yang, Amr H. A. Abdallah, and Markus A. Lill. *Calculation of Thermodynamic Properties of Bound Water Molecules*, pages 389–402. Springer New York, New York, NY, 2018.

[4] Matthew R. Masters, Yang Ying Mahmoud, Amr H., and Markus A. Lill. Efficient and accurate hydration site profiling for enclosed binding sites. *J. Chem. Inf. Model.*, 58(11):2183–2188, 2018. PMID: 30289252.

[5] Amr H. Mahmoud, Matthew R. Masters, Ying Yang, and Markus A. Lill. Elucidating the multiple roles of hydration for accurate protein-ligand binding prediction via deep learning. *Communications Chemistry*, 3(1), February 2020.

[6] Bingjie Hu and Markus A. Lill. WATsite: Hydration site prediction program with PyMOL interface. *J. Comput. Chem.*, 35(16):1255–1260, April 2014.

[7] Ying Yang, Bingjie Hu, and Markus A. Lill. Analysis of factors influencing hydration site prediction based on molecular dynamics simulations. *J. Chem. Inf. Model.*, 54(10):2987–2995, October 2014.
