# Supplementary material for: Instantaneous generation of protein hydration properties from static structures
Source: Commun Chem. 2020 Dec 11;3:188. doi: 10.1038/s42004-020-00435-5 (PMC9814540; doi:10.1038/s42004-020-00435-5)
Supplement: Supplementary file 2 — Description of Additional Supplementary Files [file 42004_2020_435_MOESM2_ESM.pdf]

Description of Additional Supplementary Files

File Name: Supplementary Data 1
Description: PDB ids for cross-validation sets.

File Name: Supplementary Data 2
Description: PDB ids for training set.

File Name: Supplementary Data 3
Description: PDB ids for test set
